# Constrained CPD of Complex-Valued Multi-Subject fMRI Data via Alternating Rank-*R* and Rank-1 Least Squares

**DOI:** 10.1109/TNSRE.2022.3198679

**Published:** 2022-09-19

**Authors:** Li-Dan Kuang, Qiu-Hua Lin, Xiao-Feng Gong, Jianming Zhang, Wenjun Li, Feng Li, Vince D. Calhoun

**Affiliations:** School of Computer and Communication Engineering, Changsha University of Science and Technology, Changsha 410114, China; School of Information and Communication Engineering, Dalian University of Technology, Dalian 116024, China; School of Information and Communication Engineering, Dalian University of Technology, Dalian 116024, China; School of Computer and Communication Engineering, Changsha University of Science and Technology, Changsha 410114, China; School of Computer and Communication Engineering, Changsha University of Science and Technology, Changsha 410114, China; School of Computer and Communication Engineering, Changsha University of Science and Technology, Changsha 410114, China; Tri-Institutional Center for Translational Research in Neuroimaging and Data Science (TReNDS), Georgia State University, Atlanta, GA 30302 USA, and also with the Georgia Institute of Technology, Emory University, Atlanta, GA 30322 USA

**Keywords:** Canonical polyadic decomposition (CPD), complex-valued fMRI data, orthonormality, shift-invariance, source phase sparsity

## Abstract

Complex-valued shift-invariant canonical polyadic decomposition (CPD) under a spatial phase sparsity constraint (pcsCPD) shows excellent separation performance when applied to band-pass filtered complex-valued multi-subject fMRI data. However, some useful information may also be eliminated when using a band-pass filter to suppress unwanted noise. As such, we propose an alternating rank-*R* and rank-1 least squares optimization to relax the CPD model. Based upon this optimization method, we present a novel constrained CPD algorithm with temporal shift-invariance and spatial sparsity and orthonormality constraints. More specifically, four steps are conducted until convergence for each iteration of the proposed algorithm: 1) use rank-*R* least-squares fit under spatial phase sparsity constraint to update shared spatial maps after phase de-ambiguity; 2) use orthonormality constraint to minimize the cross-talk between shared spatial maps; 3) update the aggregating mixing matrix using rank-*R* least-squares fit; 4) utilize shift-invariant rank-1 least-squares on a series of rank-1 matrices reconstructed by each column of the aggregating mixing matrix to update shared time courses, and subject-specific time delays and intensities. The experimental results of simulated and actual complex-valued fMRI data show that the proposed algorithm improves the estimates for task-related sensorimotor and auditory networks, compared to pcsCPD and tensorial spatial ICA. The proposed alternating rank-*R* and rank-1 least squares optimization is also flexible to improve CPD-related algorithm using alternating least squares.

## I. Introduction

Tensor decomposition of multi-subject functional magnetic resonance imaging (fMRI) data not only can retain multiway linkages and interactions, but also can extract common spatiotemporal information which can be used for studying the brain function and brain disease diagnose [1], [2], [3], [4], [5]. Canonical polyadic decomposition (CPD), one of the typical tensor decomposition methods, has been increasingly applied to multi-subject fMRI data. Different from matrix methods such as independent component analysis (ICA) [6], [7], negative matrix decomposition and dictionary learning [8], [9], [10], [11], [12], CPD can well retain the high-order structure information of multi-subject fMRI data and preserve the uniqueness under some mild conditions [13]. Generally speaking, CPD treats multi-subject fMRI data as a three-way tensor in terms of spatial, temporal and subject modes [14], and decomposes fMRI data as shared spatial maps (SMs), shared time courses (TCs), and subject-specific intensities [15], [16], [17]. However, the complex- valued multi-subject fMRI data inevitably inherit high noisy nature and high inter-subject spatiotemporal variability. Therefore, the complex-valued multi-subject fMRI data do not well conform pure CPD model, and CPD without constraint shows unsatisfying separation performance. With regard to inter-subject temporal variability caused by the hemodynamic delay, Mørup *et al.* proposed a shift-invariant CPD by incorporating time delays into the CPD model [18]. Based on an alternating least squares (ALS) updating rule, the shift-invariant CPD utilizes a frequency-domain method to estimate time delays and shared TCs, which costs less computation time and is more accurate than the existing time-domain exhaustive searching strategy [18].

Complex-valued fMRI data have been demonstrated to contain additional useful information beyond the typically-used magnitude-only fMRI data [6], [17]. In addition, the SMs extracted from complex-valued fMRI data inherit the small spatial source phase characteristic. More specifically, the phase values of blood oxygenation level dependent (BOLD) -related voxels focus on small values in the range of [−4/*π,* 4/*π*], while unwanted voxels tend to have large phase values in the range of [−*π,* −4/*π*) and (4/*π, π*] [6], [17]. Based on this small spatial source phase characteristic, Kuang *et al.* proposed a complex-valued shift-invariant CPD with a phase sparsity constraint (shorted as pcsCPD) [17]. The pcsCPD first updates shared SMs, shared TCs, subject-specific time delays and subject intensities using ALS, and second updates the shared SMs based on the smoothed *λ*_0_ norm model of phase sparsity constraint. The pcsCPD obviously outperformed the complex-valued shift-invariant CPD, tensorial spatial independent component analysis (T-sICA) and CPD when applied to the complex-valued multi-subject fMRI data with a band-pass filter [17]. However, some useful low- and high-frequency information may be lost by using the filtered fMRI data.

Besides the small spatial source phase characteristic, some studies incorporated spatial independence into CPD [13], [15], [16], [20], [21], [22], such as popular T-sICA [15], CPD based on a single mode blind source separation [13] and a tensorial decomposition method of combining ICA and shift-invariant CPD (shorted as ICA-sCPD) [16]. These methods first extracted shared SMs and aggregating mixing matrix using widely-used spatial ICA [13], [15], [16], [20], [21], [22]. Subsequently, these methods second obtained the shared TCs, subject-specific time delays and intensities (taking ICA-sCPD for example) by rank-1 ALS on a series of rank-1 matrices that were constructed by each column of the aggregating mixing matrix. Due to relax the strict CPD model of multi-subject fMRI data using rank-*R* ICA (*R* is number of components) and rank-1 ALS, ICA-sCPD and T-sICA obviously achieved better separation performance than shift-invariant CPD and CPD which only exploited rank-*R* ALS. However, these methods relied on ICA and could not fully take advantage of structure information of three-way tensor due to different cost functions of ICA and rank-1 ALS.

In addition, the spatial orthogonality among components has been taken into account in some studies. The spatial orthogonality constraint has been verified reducing the correlation and cross-talk between SM components and discard the unimportant information [16], [23], [24]. Above all, our contributions are as follows:

1. We first propose a new alternating update rule that combines rank-*R* least square to update shared SMs and a series of rank-1 least squares to update shared TCs and subject intensities, named as AR_*R*_R_1_LS. The proposed AR_*R*_R_1_LS that aims at minimizing the error squares of CPD model, can relax the strict CPD models and outperforms several state-of-the-art CPD methods.
2. Based on AR_*R*_R_1_LS, we propose a novel constrained CPD with shift-invariance and spatial phase sparsity and orthonormality constraints (shorted as sAR_*R*_R_1_LS-PO), to comprehensively alleviate the high noise nature and high spatiotemporal of complex-valued multi-subject fMRI data.
3. Complex-valued finger-tapping fMRI data experiments verify that the proposed sAR_*R*_R_1_LS-PO method can well extract task-related sensorimotor and auditory components not only in raw fMRI data analysis but also in filtered fMRI data analysis. Besides, sAR_*R*_R_1_LS-PO gets better task-related shared SM and TC estimates in intact but noisier raw fMRI data analysis than in incomplete filtered fMRI data. This indicates that some meaningful information is lost by the band-pass filter strategy.

We organize our paper as follows. We firstly describe details of our proposed AR_*R*_R_1_LS and sAR_*R*_R_1_LS-PO methods in Section II. We secondly introduce the simulated and experimental fMRI datasets as well as the performance indices for algorithm evaluation in Section III. We thirdly give results of simulated and experimental fMRI data experiments to show the advantage of our proposed methods in Section IV. We lastly have conclusion and discussion in Section V.

## II. The Proposed Methods

### Notations:

We denote scalar, matrix, and tensor as italic lower-case letter (e.g., *a*), bold lower-case letter (e.g., **a**), bold upper-case letter (e.g., **A**), and underlined bold capital letter (e.g., $\underline{A}$), respectively. We here assume $\underline{X}=\left\{x_{v,j,k}\right\}\in {\mathbb{C}}^{V\times J\times K}$ as three-way (voxel × time × subject) multi-subject fMRI data, $S≜\left[s_{1},\cdots ,s_{R}\right]=\left\{s_{v,r}\right\}\in {\mathbb{C}}^{V\times R}$ as shared SMs, $B≜\left[b_{1},\cdots ,b_{R}\right]=\left\{b_{j,r}\right\}\in {\mathbb{C}}^{J\times R}$ as shared TCs, $𝒯≜\left[\tau_{1},\cdots ,\tau_{R}\right]=\left\{\tau_{k,r}\right\}\in {\mathbb{R}}^{K\times R}$ as subject-specific time delays and $C≜\left[c_{1},\cdots ,c_{R}\right]=\left\{c_{k,r}\right\}\in {\mathbb{C}}^{K\times R}$ as subject-specific intensities, where *V* is the number of in-brain voxels, *J* is the number of time points, *K* is the number of subjects, *R* is the number of components, and *v* = 1, . . . , *V*, *j* = 1, . . . , *J*, *k* = 1, . . . , *K*, *r* = 1, . . . , *R*. $X^{\left(k\right)}\in {\mathbb{C}}^{V\times J}(k=1,\ldots ,K)$ denotes the fMRI data of subject *k*. $X_{\left(1\right)}\in {\mathbb{C}}^{V\times JK}$, $X_{\left(2\right)}\in {\mathbb{C}}^{J\times VK}$ and $X_{\left(3\right)}\in {\mathbb{C}}^{K\times VJ}$ are the mode-1, mode-2, mode-3 matrices of $\underline{X}$. ⨀ is the Khatri-Rao product. Re{·}, Im{·}, |·|, and *θ*{·} are the real, imaginary, magnitude and angle parts of a complex-valued variable.

In this section, we first introduce CPD model of multi-subject fMRI data, second present the AR_*R*_R_1_LS updating rule, and then narrate the detailed procedure of sAR_*R*_R_1_LS-PO.

### A. CPD Model of Multi-Subject fMRI Data

We assume the three-way complex-valued multi-subject fMRI data $\underline{X}$ conforms to the CPD model as follows:

(1) $$\underline{X}=\sum_{r=1}^{R}s_{r}\circ b_{r}\circ \;c_{r}+\underline{E}.$$

CPD decomposes $\underline{X}$ into *R* rank-1 tensors and each rank-1 tensor includes the outer product of shared SMs $s_{r}=\left\{s_{v,r}\right\}\in {\mathbb{C}}^{V}$, shared TCs $b_{r}=\left\{b_{j,r}\right\}\in {\mathbb{C}}^{J}$, and subject intensities $c_{r}=\left\{c_{k,r}\right\}\in {\mathbb{C}}^{K}$. Calculating *R* is a NP-hard problem [25]. The classical ALS alternately estimates **s**_*r*_, **b**_*r*_, and **c**_*r*_ by minimizing the following error squares:

(2) $$\underset{S,B,C}{\min}\sqrt{{\sum_{v,j,k}\left|x_{v,j,k}-\sum_{r=1}^{R}s_{v,r}b_{j,r}c_{k,r}\right|}^{2}}/VJK.$$

Thus shared SMs **S**, shared TCs **B**, and subject intensities **C** are alternatively updated until convergence as follows:

(3) $$S\leftarrow X_{\left(1\right)}{\left(C⊙B\right)}^{†T},$$

(4) $$B\leftarrow X_{\left(2\right)}{\left(C⊙S\right)}^{†T},$$

(5) $$C\leftarrow X_{\left(3\right)}{\left(B⊙S\right)}^{†T}.$$

### B. Alternating Rank-R and Rank-1 Least Squares (AR_R_R_1_LS)

Based on CPD model given in (1), the proposed AR_*R*_R_1_LS firstly updates shared SMs **S** using (3). Secondly, AR_*R*_R_1_LS updates the aggregating mixing matrix $M=C⊙B\in {\mathbb{C}}^{JK\times R}$ which concludes the information of **B** and **C** using **S** by a rank-*R* least-square fit:

(6) $$M\leftarrow {\left(S^{†}X_{\left(1\right)}\right)}^{T}.$$

Subsequently, each column vector of **M** denoted as $m_{r}\in {\mathbb{C}}^{JK\times 1}$ can be transformed as a rank-1 matrix $M_{r}\in {\mathbb{C}}^{J\times K}$ according to the following rule:

(7) $$M_{r}=[m_{r}(1:J),\cdots ,m_{r}((k-1)J+1:kJ),\cdots ,\;\;m_{r}((K-1)J+1:KJ)],$$

where **m**_*r*_((*k*−1)*J* +1 : *kJ*) is the vector with the ((*k*−1)*J* + 1)th to *k J* th elements of **m**_*r*_, *k* = 1,· · · , *K*. The **M**_*r*_ can be written as:

(8) $$M_{r}=b_{r}c_{r}^{T}+E_{r},$$

where **E**_*r*_ is the crosstalk from other components. The AR_*R*_R_1_LS then updates **b**_*r*_ and **c**_*r*_ by rank-1 least-square fits based on **M**_*r*_ (*r* = 1, . . . , *R*), and we have:

(9) $$\{\begin{array}{l} b_{r}\leftarrow M_{r}c_{r}^{T†},\\ c_{r}\leftarrow M_{r}b_{r}^{T†}. \end{array}$$

The AR_*R*_R_1_LS iteratively updates **S** based on (1) and **B** and **C** using (9) until convergence. Due to utilizing the rank-1 least-square fit, the simple but efficient AR_*R*_R_1_LS not only relaxes the compact CPD model but also is much faster than classical ALS.

### C. Proposed Constrained CPD Based on AR_R_R_1_LS

Based upon AR_*R*_R_1_LS updating rule and shift-invariant CPD model, we propose a constrained CPD by incorporating the spatial phase sparsity and spatial othonormality constraints into shift-invariant CPD, shorted as sAR_*R*_R_1_LS-PO, to decompose complex-valued multi-subject fMRI data with high noisy and high spatiotemporal variability.

#### 1) The Cost Function:

The proposed sAR_*R*_R_1_LS-PO assumes the complex-valued multi-subject fMRI data conforms to the shift-invariant CPD model as follows [17]:

(10) $$x_{v,j,k}=\sum_{r=1}^{R}s_{v,r}b_{r}\left(j-\tau_{k,r}\right)c_{k,r}+e_{v,j,k},$$

where *b*_*r*_(*j* − *τ*_*k,r*_) denotes *b*_*j,r*_ with time delay *τ*_*k,r*_. More specifically, $b_{r}^{\left(k\right)}={\left[b_{r}\left(1-\tau_{k,r}\right),\cdots ,b_{r}\left(J-\tau_{k,r}\right)\right]}^{T}$ is obtained by cyclic left shifting **b**_*r*_ with *τ*_*k*_,_*r*_ points if *τ*_*k*_,_*r*_ > 0, otherwise cyclic right shifting **b**_*r*_ with *τ*_*k*_,_*r*_ points. $B^{\left(k\right)}≜\left[b_{1}^{\left(k\right)},\cdots ,b_{R}^{\left(k\right)}\right]=\left\{b_{r}\left(j-\tau_{k,r}\right)\right\}\in {\mathbb{C}}^{J\times R}$ denotes the TCs of subject *k*. $\underline{E}=\left\{e_{v,j,k}\right\}\in {\mathbb{C}}^{V\times J\times K}$ is the residual tensor.

Aiming at exploiting the small phase characteristic of shared SMs and eliminating the crosstalk between shared SMs, sAR_*R*_R_1_LS-PO additionally incorporates the spatial phase sparsity and orthonormality constraints into complex-valued shift-invariant CPD model. As a whole, sAR_*R*_R_1_LS-PO iteratively updates the shared SMs **S**, the shared TCs **B**, the subject-specific time delays 𝒯 and intensities **C** by minimizing the following cost function until convergence:

(11) $$\left\{ \begin{array}{l} \underset{S,B,𝒯,C}{\arg\min}\sum_{v,j,k}\|x_{v,j,k}-\sum_{r=1}^{R}s_{v,r}b_{r}\left(j-\tau_{k,r}\right)c_{k,r}\|/VJK\\ +\lambda \sum_{v=1}^{V}\sum_{r=1}^{R}f_{\sigma }\left\{\left|s_{v},r\right|,\theta \left(s_{v},r\right)\right\}/V\\ \;s.t.\;S^{T}S=I \end{array}\right.$$

where $\sum_{v=1}^{V}\sum_{r=1}^{R}f_{\sigma }\left\{\left|s_{v},r\right|,\theta \left(s_{v},r\right)\right\}$ is a smoothed *λ*_0_ norm regularization to minimize the large-phase values of **S**, which is defined as [17]:

(12) $$f_{\sigma }\left\{\left|s_{v,r}\right|,\theta \left(s_{v,r}\right)\right\}\;\;=\left\{ \begin{array}{l} 1-\exp\left\{-{\left|s_{v,r}\right|}^{2}/2\sigma^{2}\right\},\;\left|\theta \left(s_{v,r}\right)\right|\geq \theta_{r}^{th}\\ 1,\;\left|\theta \left(s_{v,r}\right)\right|<\theta_{r}^{th} \end{array}\right.$$

where exp{·} is the exponential function and *θ*(*s*_*v,r*_) denotes the phase value of phase-corrected *s*_*v,r*_ and $\theta_{r}^{th}$ is a threshold of |*θ*(*s*_*v,r*_)| ∈ [0, *π*] for adding sparsity constraint on the unwanted voxels. In order to retain more BOLD-related voxels, $\theta_{r}^{th}$ is defined to segment the largest *V*/3 values of |*θ*(*s*_*v,r*_)| as suggested in [17].

#### 2) First Update of S Based on Rank-R Least-Square Fit Under a Phase Sparsity Constraint:

In order to reduce voxels with large phase values of shared SMs **S**, we obtain phase-corrected shared SMs by an accurate phase ambiguity based on the maximal correlation coefficient between the phase-rotated shared SM component and its magnitude, and then present the first update rule of **S** based on rank-*R* least-square fit with spatial phase sparsity constraint.

Firstly, we obtain the current shared SMs by **S** = **X**_(1)_ **M**^†*T*^. The aggregating mixing matrix **M** includes the information of shared TCs, subject-specific time delays, and intensities, and equals to

(13) $$M=\left[\begin{array}{cccc} b_{1}^{\left(1\right)}c_{1,1} & b_{2}^{\left(1\right)}c_{1,2} & \cdots & b_{R}^{\left(1\right)}c_{1,R}\\ b_{1}^{\left(2\right)}c_{2,1} & b_{2}^{\left(2\right)}c_{2,2} & \cdots & b_{R}^{\left(2\right)}c_{2,R}\\ \vdots & \vdots & \ddots & \vdots \\ b_{1}^{\left(K\right)}c_{K,1} & b_{2}^{\left(K\right)}c_{K,2} & \cdots & b_{R}^{\left(K\right)}c_{K,R} \end{array}\right].$$

We then perform phase de-ambiguity on each shared SM estimate **s**_*r*_(*r* = 1, . . . , *R*) based on the maximal correlation coefficient between the phase-rotated shared SM component and its magnitude |**s**_*r*_|:

(14) $${\hat{\theta }}_{r}=\arg\underset{0\leq \theta \leq 2\pi }{\max}{\left\|\mathrm{corr}\left\{\exp\left\{i\theta_{r}\right\}s_{r}\cdot \mathrm{BM}(\pm \pi /4),\left|s_{r}\right|\right\}\right\|}_{2},$$

where ${\hat{\theta }}_{r}$ is the final rotation angle, “corr{·}” is correlation computation and BM(±*π*/4) is the binary mask with phase range [−4/*π*, 4/*π*] which can be generated as [19]:

(15) $$\text{BM}(\pm \pi /4)=\{\begin{array}{ll} 1, & \;\text{if}\;\theta \left(s_{v},r\right)\in [-4/\pi ,4/\pi ],\\ 0, & \;\text{otherwise}.\; \end{array}$$

By masking the phase-corrected exp{*iθ*_*r*_}**s**_*r*_ with BM(±*π*/4), the de-noised SM can be achieved. We here let *θ*_*r*_ = 2*π*/*k*, *k* = 1, . . . *K*, so there are *K* rotation angles for detecting, i.e., 2*π*/*K*, · · ·, 2*π*. The lager *K* is, the more accurate the detected rotation angle ${\hat{\theta }}_{r}$ is. We set *K* = 128 in this paper. Therefore, the phase after de-ambiguity for **s**_*r*_ is $\theta \left(s_{r}\right)=\theta \left(\exp\left\{i{\hat{\theta }}_{r}\right\}s_{r}\right)$.

According to the cost function of the proposed method (i.e., (11)), we use the steepest descent method to first update the shared SMs **S** with phase sparsity constraint:

(16) $$S\leftarrow X_{\left(1\right)}M^{†T}-\lambda \Delta S{\left(M^{H}M\right)}^{-1},$$

and $\Delta S=\left\{\Delta s_{v,r}\right\}\in {\mathbb{C}}^{V\times R}$ can be calculated as follows [17]:

(17) $$\Delta s_{v,r}=\exp\left\{\theta \left(s_{v,r}\right)\right\}f_{\sigma }^{\prime }\left\{\left|s_{v,r}\right|,\theta \left(s_{v,r}\right)\right\},$$

where $f_{\sigma }^{\prime }\left\{\left|s_{v,r}\right|,\theta \left(s_{v,r}\right)\right\}$ is the derivative of *f*_*σ*_{|*s*_*v,r*_|, *θ*(*s*_*v,r*_)} equaling to [17]:

(18) $$f_{\sigma }^{\prime }\left\{\left|s_{v,r}\right|,\theta \left(s_{v,r}\right)\right\}\;\;=\{\begin{array}{ll} \frac{\left|s_{v,r}\right|}{\sigma^{2}}\exp\left\{-\frac{{\left|s_{v,r}\right|}^{2}}{\sigma^{2}}\right\}, & \left|\theta \left(s_{v,r}\right)\right|\geq \theta_{r}^{th},\\ 0, & \left|\theta \left(s_{v,r}\right)\right|<\theta_{r}^{th}. \end{array}$$

The parameter *σ* should be slowly decreased to escape from local minima and singular values [26], [27], thus we here let $\sigma_{\text{iter}\;}=\gamma \sigma_{\text{iter-1}}$, where ‘iter’ is the iteration index, and *γ* is the decrease rate, and 0.9 < *γ* < 1 (we set *γ* = 0.99 here).

#### 3) Second Update of S by Spatial Orthonormality Constraint:

We secondly update the shared SMs **S** by adding the spatial orthonormality constraint. We can perform the economical singular value decomposition (SVD) on **S**, and obtain orthonormal **S** based on the following computations:

(19) $$\{\begin{array}{l} {}[U,\Sigma ,D]=\mathrm{svd}(S),\\ S\leftarrow UD^{H}, \end{array}$$

where svd(·) is economical SVD which decomposes **S** into a left singular matrix $U\in {\mathbb{C}}^{V\times R}$, a diagonal matrix $\Sigma \in {\mathbb{C}}^{R\times R}$ and right singular matrix $D\in {\mathbb{C}}^{R\times R}$.

#### 4) Updates of B, 𝒯, and C by Complex-Valued Shift-Invariant Rank-1 Least-Square Fit:

After obtaining the shared SMs, we can update the aggregating mixing matrix **M** based on rank-*R* least-square fit using (6), and obtain a series of rank-1 matrices **M**_*r*_ based on (7). We then perform the complex-valued shift-invariant rank-1 least-square fit on **M**_*r*_, and have

(20) $$m_{r,k}=b_{r}^{\left(k\right)}c_{k,r}+e_{r}^{\left(k\right)},$$

where **m**_*r*_,_*k*_ is the *k*th column vector of **M**_*r*_. We can have the following updates of **b**_*r*_ and **c**_*r*_ based on shift-invariant rank-1 least-square fit by minimizing the norm of $e_{r}^{\left(k\right)}$ [16]:

(21) $$\left\{ \begin{array}{l} {\tilde{b}}_{r}\leftarrow {\tilde{M}}_{r}{\left(c_{r}\cdot \exp\left\{-i2\pi \frac{f-1}{J}\tau_{r}\right\}\right)}^{T†},\\ \;b_{r}\leftarrow b_{r}/\left\|b_{r}\right\|,\\ c_{k,r}\leftarrow {\left(m_{r,k}\right)}^{T}{\left(b_{r}^{\left(k\right)}\right)}^{T†},\\ k=1,\ldots ,K,\;\text{and}\;c_{r}\leftarrow c_{r}/\left\|c_{r}\right\|, \end{array}\right.$$

where “·” is dot product, $i=\sqrt{-1}$, ${\tilde{b}}_{r}\in {\mathbb{C}}^{F}$ and ${\tilde{M}}_{r}\in {\mathbb{C}}^{F\times K}$ are the frequency-domain forms of **b**_*r*_ and **M**_*r*_.

For the update of ***τ***_*r*_, the minimization of the least-square error ${\left\|m_{r,k}-b_{r}^{\left(k\right)}c_{k,r}\right\|}^{2}$ in (20) can be expanded as

(22) argminτk,r‖mr,k−br(k)ck,r‖2=argminτk,r{‖mr,k‖2+‖br(k)ck,r‖2−2Re{mr,kT}Re{br(k)ck,r}−2Im{mr,kT}Im{br(k)ck,r}}

As **m**_*r,k*_ and $b_{r}^{\left(k\right)}$ are cyclic shifted based on the time delay *τ*_*k*_,_*r*_, the first and second terms in (22) do not vary with *τ*_*k*_,_*r*_. As such, the update of *τ*_*k*_,_*r*_ maximizes the sum of the third and fourth terms in (22) as follows:

(23) argmaxτk,r[2Re{mr,kT}Re{br(k)ck,r}+2Im{mr,kT}Im{br(k)ck,r}].

We further expand (23) according to supplementary materials, and thus the *τ*_*k,r*_ can be updated by:

(24) $${\hat{\tau }}_{k,r}=\underset{1\leq j\leq J}{\arg\max}\varphi_{k,r}(j),\;\tau_{k,r}={\hat{\tau }}_{k,r}-J+1,\;\;k=1,\ldots ,K.$$

For the detail derivation of (24) from (23), please see supplementary materials. The time delay *τ*_*k*_,_*r*_ in (24) is integer, which is easier and faster to estimate than non-integer time delay [18]. The shared SMs **s**_*r*_, shared TCs **b**_*r*_, subject-specific time delays τ_*r*_, and intensities **c**_*r*_ (*r* = 1, . . . , *R*) are relatively updated until convergence.

**Fig. 1.**
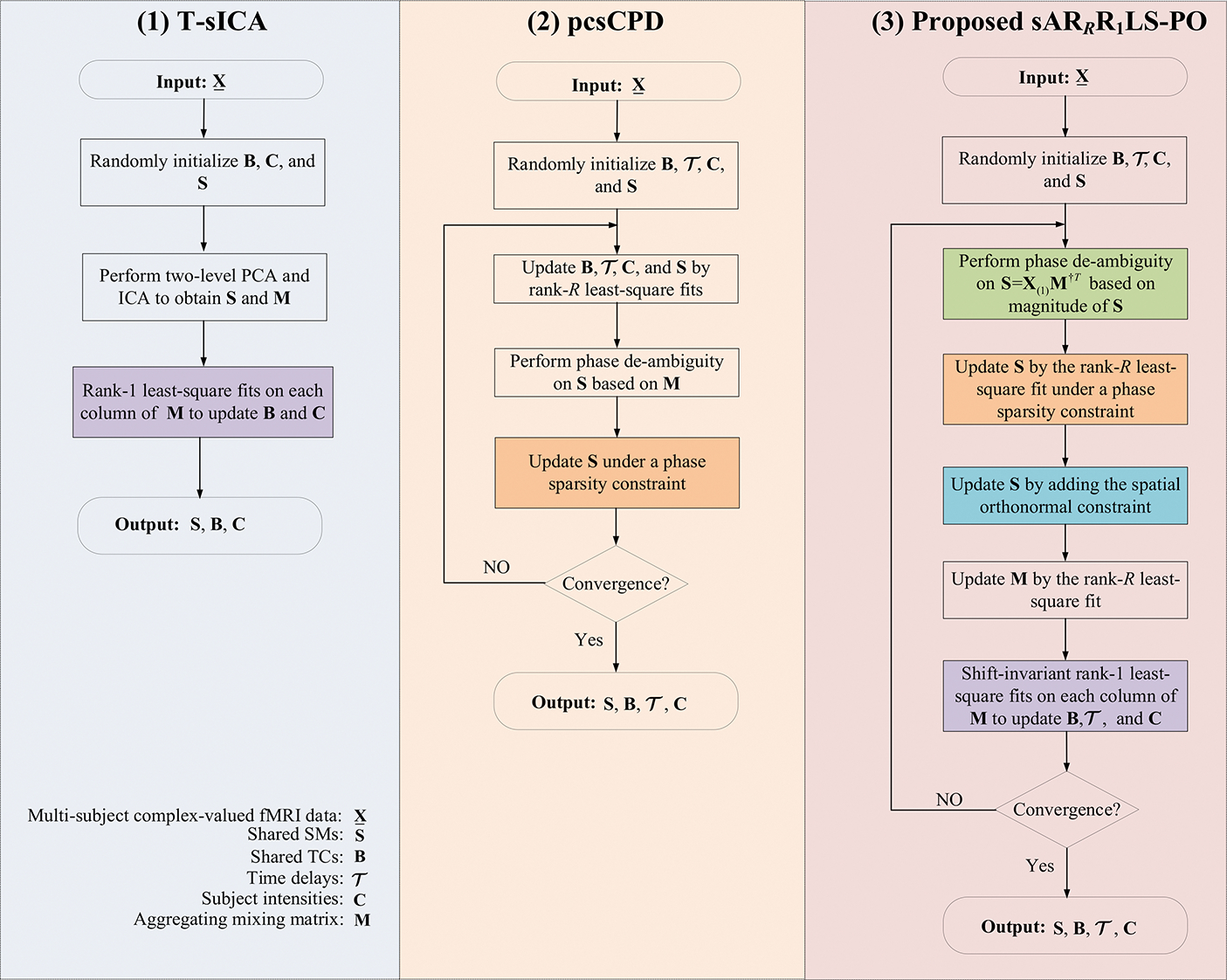
The graphical summary of T-sICA, pcsCPD, and our proposed sAR_*R*_R_1_LS-PO method.

The proposed sAR_*R*_R_1_LS-PO (the code is available in https://github.com/LidanKuang/CPD_SARRR1LS-PO) can relax CPD model, capture large spatiotemporal variabilities and avoid increasing crosstalk between components of noisy complex-valued multi-subject fMRI data. In order to show the difference of pcsCPD, T-sICA, and the proposed sAR_*R*_R_1_LS-PO, we provide a graphical summary of the three methods in Fig. 1.

## III. Experimental Methods

In order to inspect the advantage of the proposed AR_*R*_R_1_LS for decomposing multi-subject fMRI data, we compare AR_*R*_R_1_LS with several popular CPD methods for both the following simulated and experimental fMRI data analyses. The supplementary materials represent the detailed comparison results of AR_*R*_R_1_LS and several CPD methods, and show the obvious higher separation performance of AR_*R*_R_1_LS in both simulated and experimental fMRI data analyses.

Furthermore, to evaluate the efficacy of the proposed sAR_*R*_R_1_LS-PO, we compare sAR_*R*_R_1_LS-PO with T-sICA and pcsCPD by conducting both simulated and experimental complex-valued multi-subject fMRI data experiments. The widely-used complex-valued entropy bound minimization (EBM) algorithm [28] was selected for the ICA part of T-sICA. The estimated time delays range from −10~10 points. For pcsCPD and sAR_*R*_R_1_LS-PO, the parameters *σ*_0_ and *λ* are respectively set to be 2 and 4 for both simulated and experimental fMRI data as suggested in [17]. We de-noise each shared SM estimate **s**_*n*_ via the phase de-noise method in [19]. We set *ε*_iter_min_ = 10^−6^ and iter_max_ = 200 for each method. We repeat each algorithm 20 times for each case.

To show the advantage of complex-valued fMRI data analysis, we also compare our proposed method to the real-valued ICA-sCPD [16]. We also conduct real-valued ICA-sCPD on residual fMRI data after filtering to inspect the missing information. We choose real-valued Infomax algorithm [29] for ICA part of real-valued ICA-sCPD.

### A. Simulated fMRI Data

We here extend real-valued simulated multi-subject fMRI datasets in [16] into complex-valued forms. The simulated multi-subject fMRI datasets are generated by simulation toolbox SimTB at https://trendscenter.org/software/simtb/ [30], [31]. We modify SimTB to create the complex-valued fMRI datasets. For the activated voxels of SMs, we uniformly range their phase values from −*π*/18 to *π*/18 since the phase difference induced by task activation is typically less than *π*/9 [32], [33]. In contrast, the phase values of non-activated voxels for each SM range uniformly from −*π* to *π*. Each dataset contains 10 subjects, and each subject has 30 components. For each component, the SM contains 100 × 100 voxels (7688 in-brain voxels), and the TC includes 160 time points with TR = 2 seconds. Thus, after removing the out-brain voxels, the size of simulated multi-subject fMRI data is 7688 × 160 × 10. There is a task-related component for each subject as shown in Fig. 5, which is generated by a block design (40 seconds on, 30 seconds off) with a hemodynamic response function. We further change the SM activations and add shifted TCs with different time delays for each subject to simulate the spatiotemporal variability across subjects. Specifically, the SM activation changes include x and y translation changes with a uniform distribution 𝒰(−3,3) (i.e., the maximal horizontal or vertical translation 3 voxels), rotation changes were with 𝒰(−30,30) (i.e., the maximal rotation 30 degrees in a counter-clockwise or clockwise direction) and spread changes with 𝒰(1−0.12,1+0.12) (i.e., the maximal contraction or expansion 0.12). To investigate the noise effect, we also add the Gaussian noise with different signal noise ratio (SNR) levels. The SNR is defined as $20\mathrm{lg}\left({\hat{\sigma }}_{s}/{\hat{\sigma }}_{n}\right)dB$ , where ${\hat{\sigma }}_{s}$ and ${\hat{\sigma }}_{n}$ are respectively the temporal standard deviations of the source signal and Gaussian noise. We set the number of component to be 30 (i.e., ground truth) for each algorithm.

### B. Experimental fMRI Data

The experimental fMRI datasets used in our paper were collected from 16 subjects performing a finger-tapping motor task while receiving auditory instructions, and have been studied in [6], [16], [17], and [19]. All participants signed IRB-approved informed consent at the University of New Mexico. The experiments were performed on a 3T Siemens TIM Trio system with a 12-channel radio frequency (RF) coil, and used a standard Siemens gradient-echo EPI sequence to store real and imaginary data separately. The block design in the experimental paradigm is set to be alternating periods of 30 seconds on (finger tapping) and 30 seconds off (rest). Each participant was collected with 165 whole head fMRI images. We used the following parameters: field-of-view = 24 cm, slice thickness = 3.5 mm, slice gap = 1 mm, number of slices = 32, matrix size = 64 × 64, TE = 29 ms, TR = 2 s, flip angle = 70 degrees. We preprocessed the data using the SPM software package, co-registered the data using INRIAlign to compensate for movements in the fMRI time series images, and spatially normalized images into the standard Montreal Neurological Institute space. After spatial normalization, the data (originally acquired at 3.75 × 3.75 × 4.5 mm^3^) were slightly sub-sampled to 3 × 3 × 3 mm^3^, resulting in 53 × 63 × 46 voxels. Then, the images were spatially smoothed with a 10 × 10 × 10 mm^3^ full width at half-maximum Gaussian kernel. Subsequently, we also eliminated noise by performing an 8~150 mHz band-pass filter process on raw complex-valued fMRI data [17] to comprehensively investigate our proposed method and compared methods on both raw and filtered complex-valued fMRI data. When analyzing the experimental fMRI data, we show the separation performance of our proposed method for both the task-related sensorimotor and auditory components. We choose the number of components to be 50 for all methods in complex-valued experimental fMRI data analysis as suggested in [17].

### C. Performance Indices

**Fig. 2.**
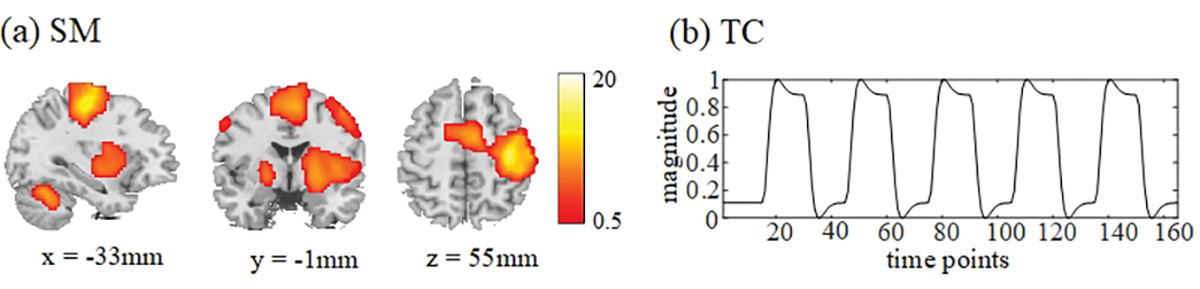
The SM and TC references of task-related sensorimotor components for experimental fMRI data. The sensorimotor SM reference (a) is the GLM map obtained from magnitude-only fMRI data. The TC references (b) are obtained by convolving the stimuli with the canonical SPM hemodynamic response functions.

For the separation performance evaluation of simulated and experimental fMRI data, we utilize the widely-used absolute Pearson correlation coefficient *ρ* between an estimate and its reference. The simulated data include references as the ground truth. For the experimental fMRI data, we use a group general linear model (GLM) map (performing one sample *t*-test on subject-specific GLM results and *p* < 0.05 for magnitude-only fMRI data) as task-related sensorimotor component in Fig. 2(a), which also has been used in [6], [16], [17], and [19]. Besides, we generate the task-related sensorimotor TC references by convolving the stimuli with the canonical SPM hemodynamic response functions in Fig. 2(b). The *ρ* values between magnitude parts of shared SMs and TCs and corresponding references are evaluated. Moreover, in order to evaluate the phase quality of SM and TC estimates, we calculate the *ρ* value between source phase mask BM(±*π*/4) of shared SM estimate and its SM reference and the *ρ* value between phase of TCs and its TC reference.

Furthermore, in order to inspect the activation of shared SMs more precisely, we denote the number of total activated voxels, the number of activated voxels inside SM reference mask, the number of activated voxels outside SM reference mask respectively as *V*_*all*_, *V*_*in*_, and *V*_*out*_ in experimental fMRI data analysis. The ratio of *V*_*in*_ and *V*_*all*_ (i.e., *V*_*in*_/*V*_*all*_) is also calculated. Higher *V*_*all*_, *V*_*in*_, and *V*_*in*_/*V*_*all*_ and lower *V*_*out*_, better separation performance.

## IV. Results

### A. Simulated fMRI Data

**Fig. 3.**
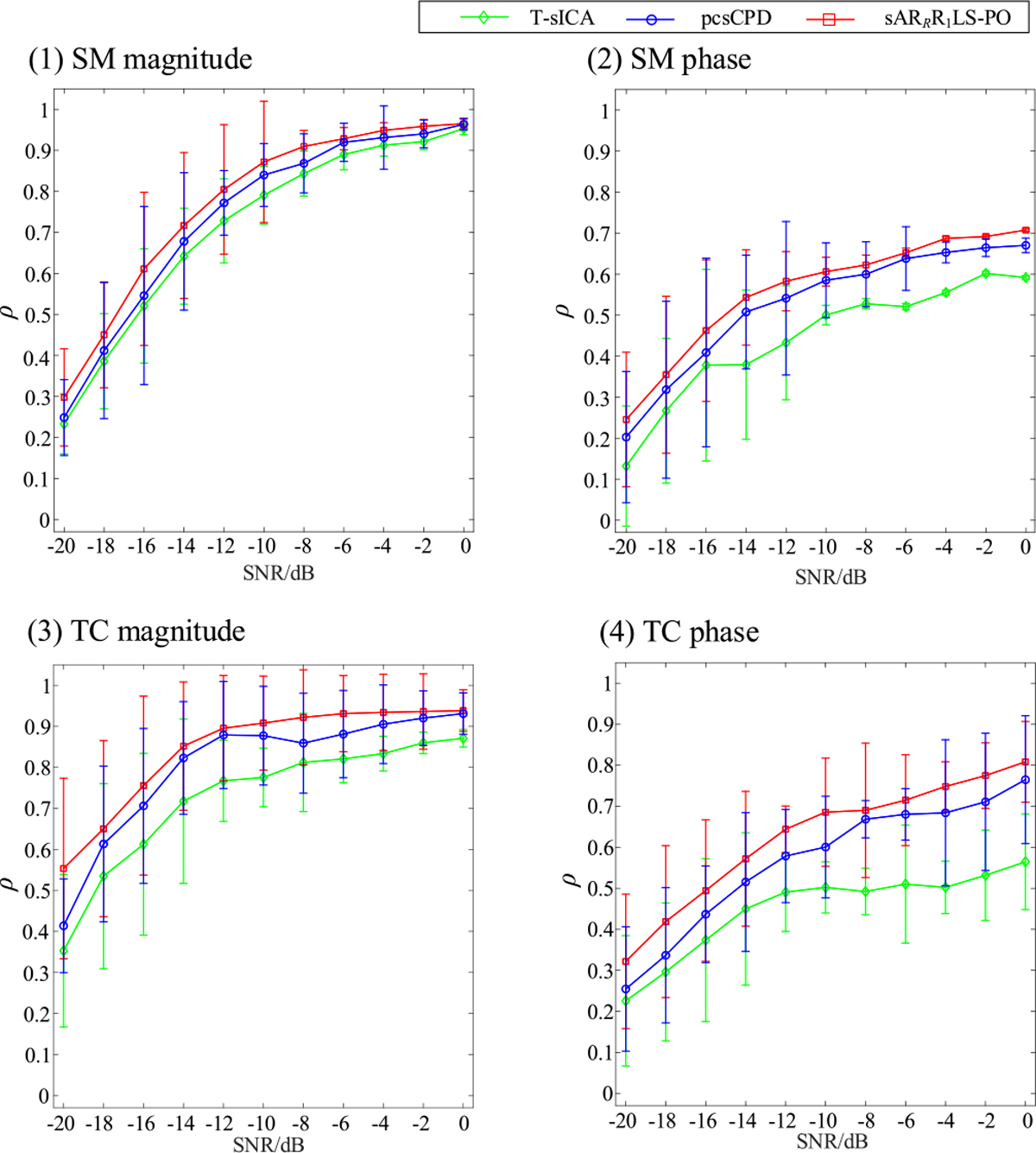
Comparison of the noise effects (from SNR = −20 dB to SNR = 0 dB with interval 2 dB) on the T-sICA, pcsCPD, and sAR_*R*_R1LS-PO in terms of the means and standard deviations of *ρ* values between the task-related shared SM magnitude (1), SM phase (2), TC magnitude (3), and TC phase (4) estimates and their ground truths. The true component number *N* = 30.

We examine noise effects of different SNR levels on T-sICA, pcsCPD, and sAR_*R*_R_1_LS-PO in simulated fMRI data analysis, as shown in Fig. 3. The proposed sAR_*R*_R_1_LS-PO has the highest average *ρ* values of magnitude and phase parts of task-related shared SMs and TCs as displayed in Fig. 3, followed by pcsCPD. Due to not considering time delays among subjects and spatial phase sparsity constraints, T-sICA ranks the last, especially for phase parts of shared SMs and TCs. The average *ρ* differences between sAR_*R*_R_1_LS-PO and other two methods are obviously larger for shared TCs than for shared SMs. The standard deviations of shared SMs and TCs for these three methods become larger when SNR levels decrease.

**Fig. 4.**
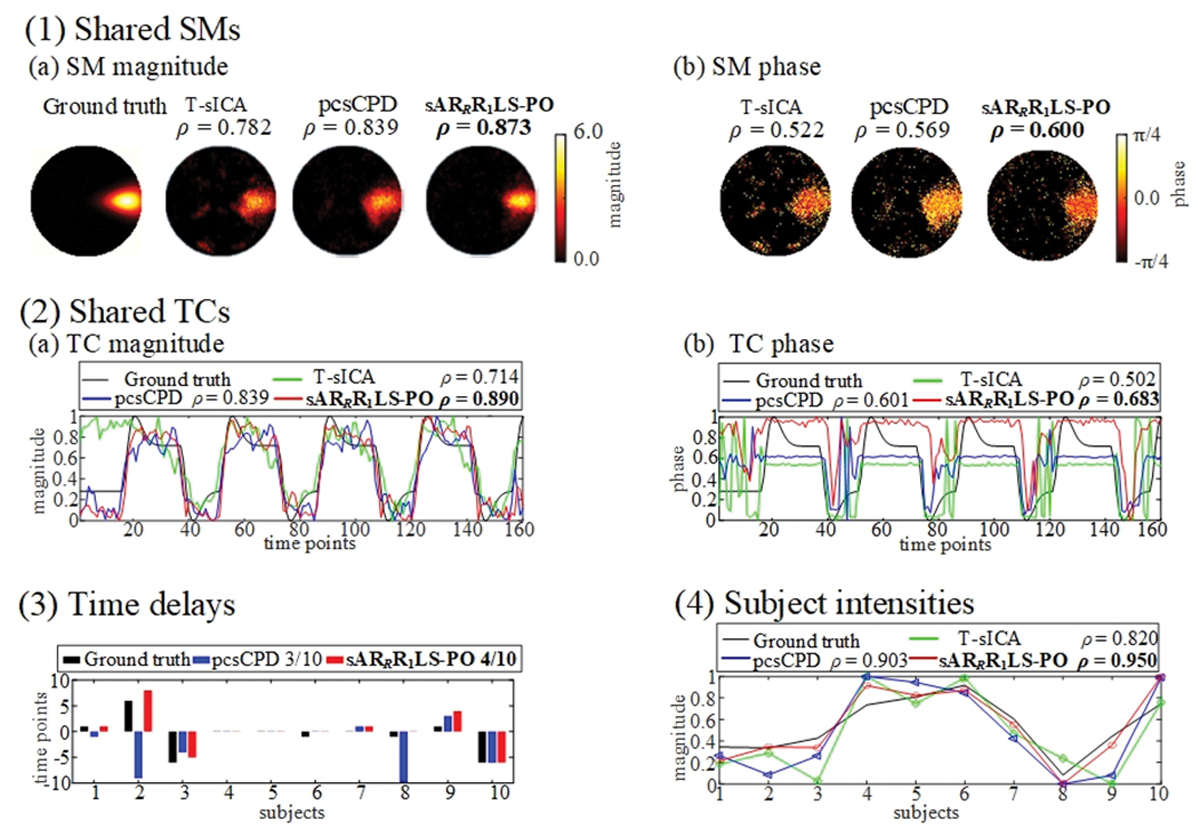
Summary of results estimated by T-sICA, pcsCPD, and sAR_*R*_R_1_LS-PO at SNR = −10 dB for simulated multi-subject fMRI data, including magnitude and phase parts of shared SMs (1), magnitude and phase parts of shared TCs (2), time delays (3), and subject intensities (4). The phase correction and de-ambiguity are performed on shared SMs, and thus the phase values of phase maps range from −*π*/4 to *π*/4.

The detailed results of typical task-related shared SMs, shared TCs, time delays, and subject intensities estimated by T-sICA, pcsCPD, and sAR_*R*_R_1_LS-PO under SNR = −10 dB are exhibited in Fig. 4. For shared SMs as displayed in Fig. 4(1), sAR_*R*_R_1_LS-PO gets not only the highest *ρ* values of magnitude and phase but also has the least crosstalk from other components. The magnitude and phase waveforms of shared TCs estimated by sAR_*R*_R_1_LS-PO also are most similar to ground truth (see Fig. 4(2)). In addition, the number of correctly estimated time delays of sAR_*R*_R_1_LS-PO is greater than that of pcsCPD (4 vs. 3), as shown in Fig. 4(3). In reality, sAR_*R*_R_1_LS-PO also has higher *ρ* value of estimated time delays and ground truth than pcsCPD (0.973 vs. 0.019). Finally, sAR_*R*_R_1_LS-PO acquires the highest *ρ* value between the magnitude of subject intensities and ground truth in Fig. 4(4), compared with T-sICA and pcsCPD.

### B. Experimental fMRI Data

In fact, the filter scheme adopted in [17] can effectively and largely reduce the noise of experimental complex-valued multi-subject fMRI data. However, some meaningful information may be lost using the filter scheme. Therefore, we evaluate separation performance of proposed sAR_*R*_R_1_LS-PO with comparison to T-sICA and pcsCPD in both experimental raw and filtered complex-valued multi-subject fMRI data analyses. The task-related sensorimotor and auditory components are analyzed. Due to limited space, the comparison results of auditory component are given in supplementary material which can further verify the evident outperformed separation performance of the proposed sAR_*R*_R_1_LS-PO in both experimental raw and filtered fMRI data analyses than T-sICA and pcsCPD.

**Fig. 5.**
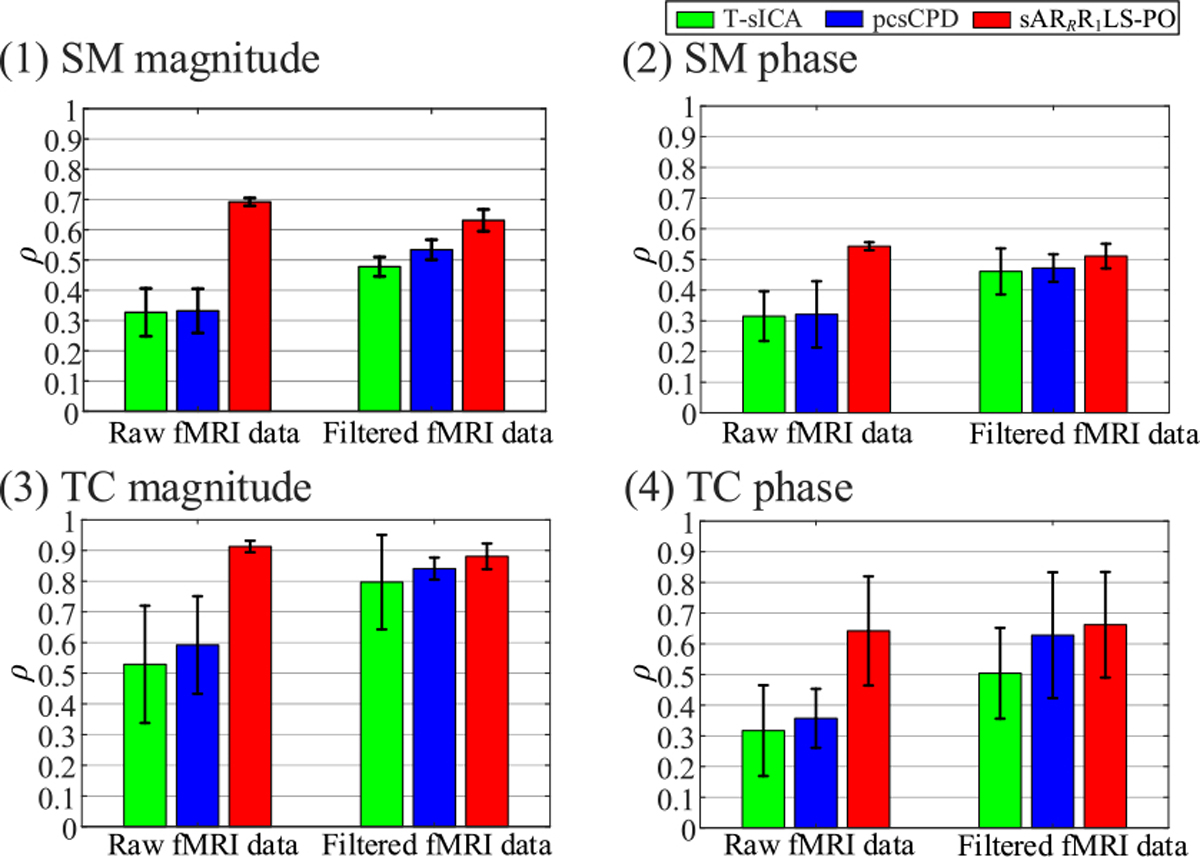
Comparison of T-sICA, pcsCPD, and sAR_*R*_R_1_LS-PO for analyzing the experimental raw and filtered complex-valued multi-subject fMRI data over 20 runs in terms of the means and standard deviations of *ρ* values for the task-related sensorimotor shared SM magnitude (1), SM phase (2), TC magnitude (3), and TC phase (4) estimates.

Fig. 5 shows the comparison of T-sICA, pcsCPD, and sAR_*R*_R_1_LS-PO in terms of means and standard deviations of *ρ* values of magnitude and phase parts of sensorimotor shared SMs and TCs in raw and filtered fMRI data analyses. The sAR_*R*_R_1_LS-PO (marked by red color) obtains the highest average *ρ* values of magnitude and phase parts of sensorimotor shared SMs and TCs in two analyses (see Fig. 5), followed by pcsCPD and T-sICA. Moreover, the gap of average *ρ* values between sAR_*R*_R_1_LS-PO and other two methods is obviously larger in raw fMRI data analysis than in filtered fMRI data analysis, under all cases as shown in Fig. 5. T-sICA and pcsCPD exhibit lower average *ρ* values of shared SMs and TCs in raw fMRI data analysis than in filtered fMRI data analysis (see Fig. 5). Meanwhile, due to incorporating the temporal shift-invariance, pcsCPD and sAR_*R*_R_1_LS-PO present obviously higher average *ρ* values of magnitude and phase parts of shared TCs than T-sICA in both two analyses as shown in Figs. 5(3)~(4). In terms of magnitude and phase parts of shared SMs and magnitude parts of shared TCs, T-sICA gets higher standard deviations of *ρ* values than pcsCPD and sAR_*R*_R_1_LS-PO, and sAR_*R*_R_1_LS-PO shows the lowest standard deviations of *ρ* values. Above results imply the better separation performance and robustness of sAR_*R*_R_1_LS-PO than pcsCPD and T-sICA for extracting sensorimotor components in both raw and filtered fMRI data analyses.

**Fig. 6.**
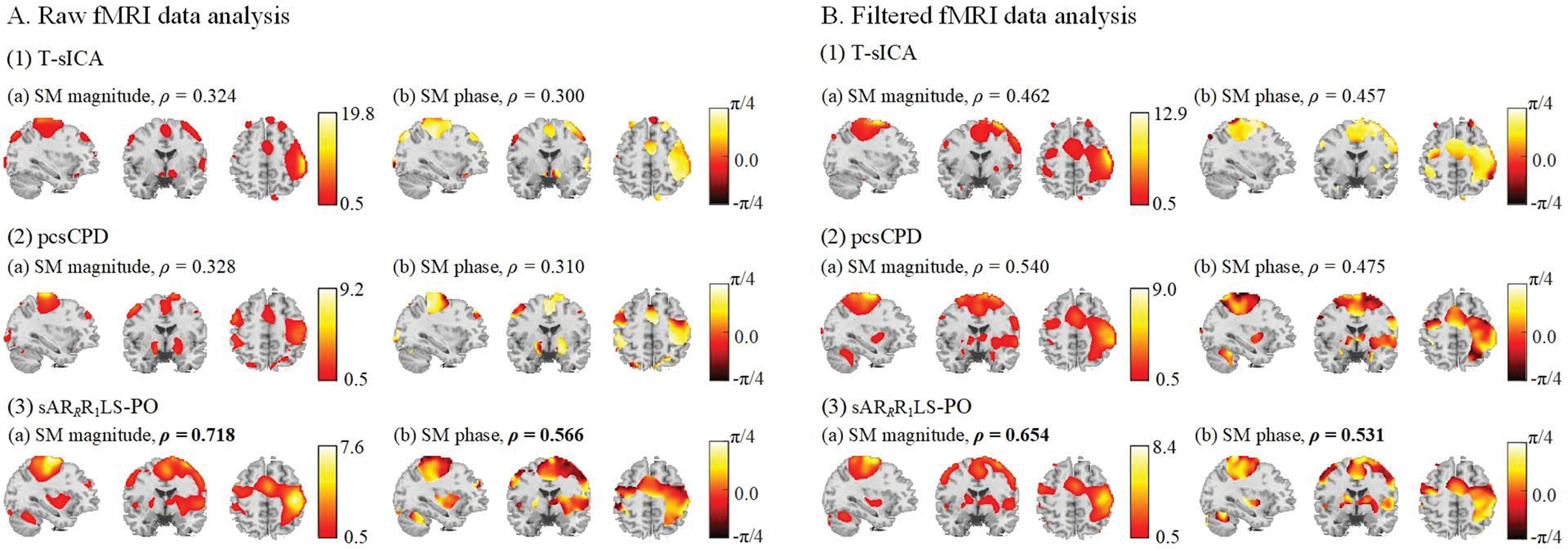
Typical task-related sensorimotor shared SMs estimated by T-sICA, pcsCPD, and sAR_*R*_R_1_LS-PO for analyzing experimental raw (A) and filtered (B) complex-valued multi-subject fMRI data. The magnitude (a) and phase images (b) of shared SMs and corresponding *ρ* values are showed. The largest *ρ* values are bold. The phase correction and de-ambiguity are performed on shared SMs, and thus the phase values of phase maps range from −*π*/4 to *π*/4.

The typical shared SMs estimated by T-sICA, pcsCPD, and sAR_*R*_R_1_LS-PO in raw and filtered fMRI data analyses are further presented in Fig. 6. The *V*_*all*_, *V*_*in*_, *V*_*out*_, and *V*_*in*_/*V*_*all*_ values of shared SM estimates are listed in Table I. In raw fMRI data analysis as shown in Fig. 6A, the shared SM estimated by sAR_*R*_R_1_LS-PO not only has the highest *ρ* values for magnitude and phase parts, but also apparently gets the largest activation region in left primary motor areas (LPMA), right primary motor areas (RPMA) and supplementary motor areas (SMA), compared with those of T-sICA and pcsCPD. The highest *V*_*all*_, *V*_*in*_, and *V*_*in*_/*V*_*all*_ values of sAR_*R*_R_1_LS-PO in Table I also reflect the highest number of task-related sensorimotor voxels for sAR_*R*_R_1_LS-PO. On the other hand, in filtered fMRI data analysis as shown in Fig. 6B, the proposed sAR_*R*_R_1_LS-PO still shows the higher *ρ* values for magnitude and phase parts of shared SM, and has higher *V*_*in*_ value (see Table I), compared with T-sICA and pcsCPD. T-sICA and pcsCPD show better shared SMs (i.e., larger spatial sensorimotor activated voxels) in filtered fMRI data analysis than in raw fMRI data analysis. This further verifies that the filter scheme can effectively reduce unwanted noise. However, on the premise of good separation performance, sAR_*R*_R_1_LS-PO extracts better shared SM estimates in raw fMRI data analysis than in filtered fMRI data analysis. This implies that some meaningful information may be lost by exploiting the filter scheme.

**Fig. 7.**
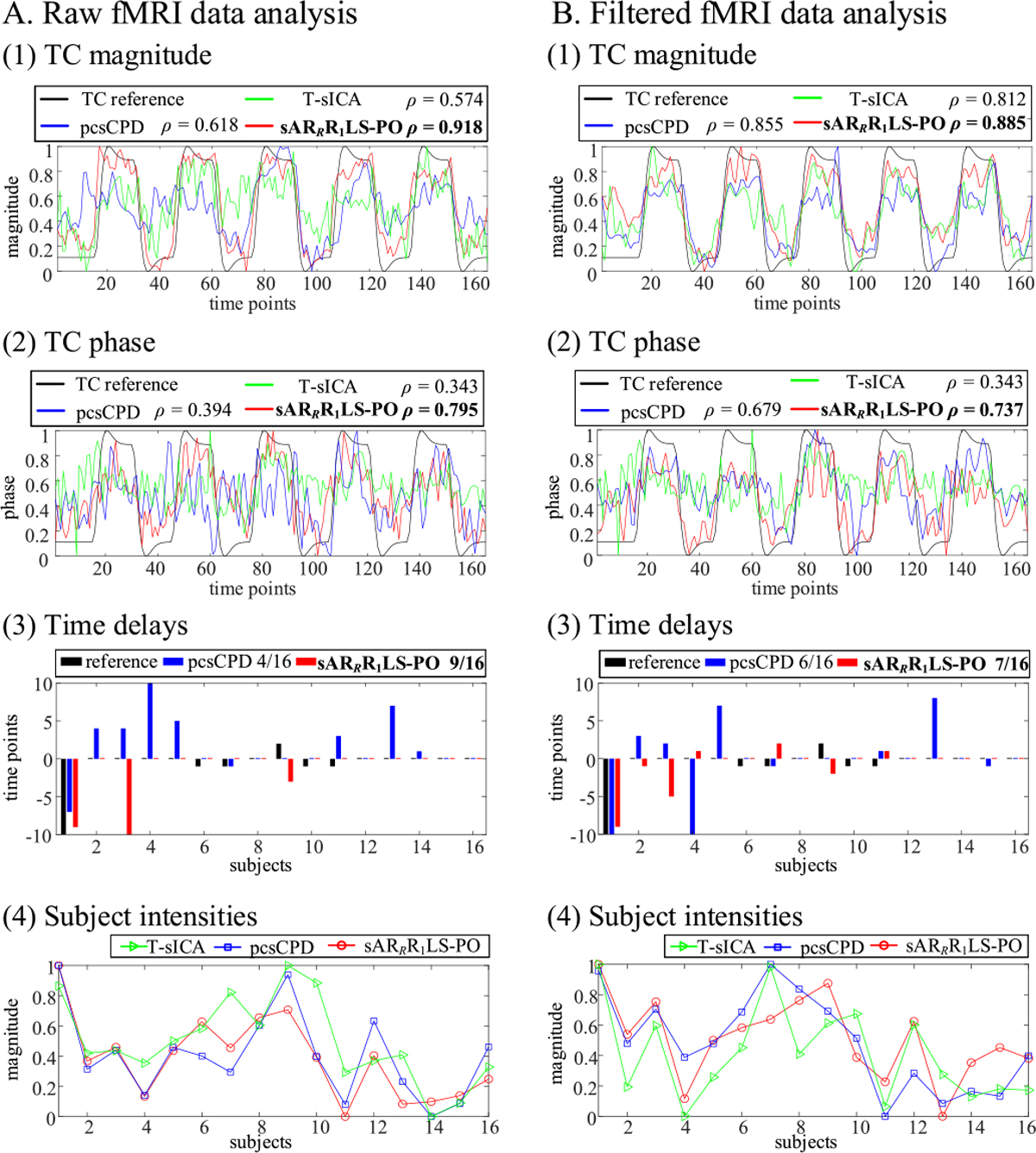
Comparison of T-sICA, pcsCPD, and sAR_*R*_R_1_LS-PO for analyzing the experimental raw (A) and filtered (B) experimental complex-valued fMRI data in terms of typical task-related sensorimotor shared TC magnitude parts (1), shared TC phase parts (2), time delays (3), and subject intensities (4). The *ρ* values of shared TC magnitude and phase parts and the number of correct time delays are calculated.

Besides sensorimotor shared SM estimates, we also exhibit Fig. 7 to comprehensively compare T-sICA, pcsCPD, and sAR_*R*_R_1_LS-PO in terms of the typical sensorimotor shared TC magnitude and phase estimates, time delay estimates, and subject intensity estimates. The sAR_*R*_R_1_LS-PO obviously extracts the sensorimotor TC magnitude and phase waveforms closest to TC reference as shown in Figs. 7(1)~(2). Consistent with results of shared SMs, T-sICA and pcsCPD have better shared TC estimates in lower-noisy filtered fMRI data analysis than in raw fMRI data analysis. While sAR_*R*_R_1_LS-PO is robust to noise (i.e., best shared TC estimates in both two analyses) and extracts better shared TC estimates for intact raw fMRI data. In addition, the inter-subject response time or hemodynamic delay differences are reflected in time delays. Compared to pcsCPD, sAR_*R*_R_1_LS-PO has higher number of correctly estimated time delays in both raw (4 vs. 9 in Fig. 7A(3)) and filtered (6 vs. 7 in Fig. 7B(3)) fMRI data analyses. Finally, for the curves of subject intensity estimates in Fig. 7(4), pcsCPD is closer to sAR_*R*_R_1_LS-PO than T-sICA in both two analyses, that is, the *ρ* values are 0.775 vs. 0.712 in raw fMRI data analysis and 0.884 vs. 0.755 in filtered fMRI data analysis.

### C. Comparison of Complex-Valued sARRR1LS-PO and the Magnitude-Only Method

**Fig. 8.**
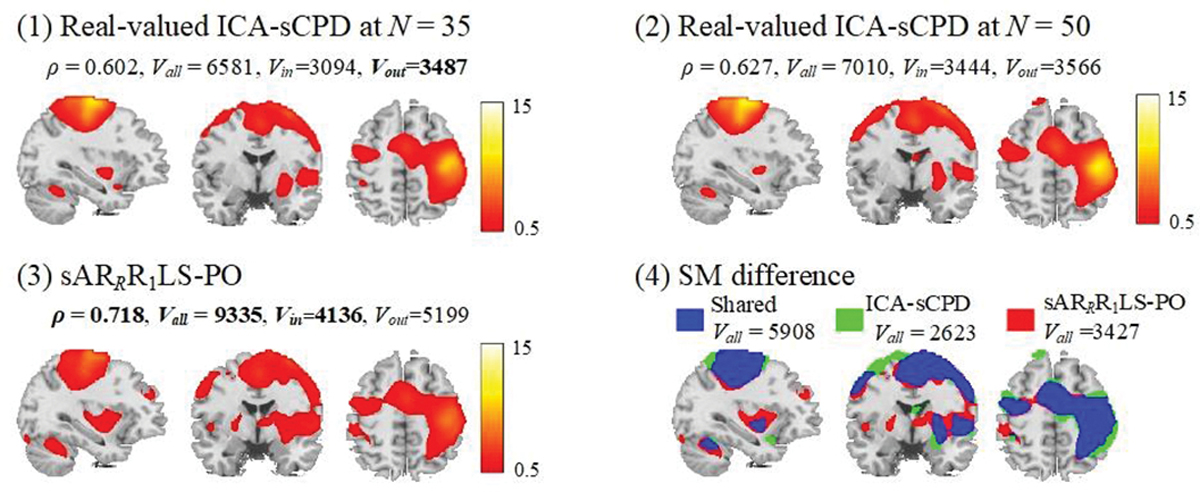
Comparison of real-valued ICA-sCPD in magnitude-only fMRI analysis and sAR_*R*_R_1_LS-PO in complex-valued fMRI analysis: (1) the shared sensorimotor SM estimated by real-valued ICA-sCPD at *N* = 35; (2) the shared sensorimotor SM estimated by real-valued ICA-sCPD at N= 50; (3) the magnitude image of shared sensorimotor SM estimated by sAR_*R*_R_1_LS-PO; (4) the SM difference between real-valued ICA-sCPD at *N* = 50 and the proposed method (the voxels detected by the both, by ICA-sCPD, or by proposed approach are respectively marked in blue, in green, and in red). The *ρ*, *V*_*all*_, *V*_*in*_, and *V*_*out*_ values are calculated.

Fig. 8 shows the comparison of the shared sensorimotor SM estimates of our proposed sAR_*R*_R_1_LS-PO and the real-valued ICA-sCPD in the magnitude-only fMRI data analysis, to verify the advantage of complex-valued fMRI data analysis. The shared SM estimates of real-valued ICA-sCPD at *N* = 35 (same with the model order in [16]) and *N* = 50 (same with the model order of the proposed method) are showed in Fig. 8. Our proposed method not only has higher *ρ* value but also gets higher *V*_*in*_, and *V*_*out*_ than real-valued ICA-sCPD at *N* = 35 and *N* = 50. The real-valued ICA-sCPD at *N* = 50 has the higher *ρ* value and the number of activated voxels than at *N* = 35. We further show the SM difference between the proposed method and two cases of real-valued ICA-sCPD, as shown in Fig. 8(4). The proposed method additionally extracts 30.7% more number of activated voxels (3427 vs. 2623) than real-valued the LPMA areas and right pallidum, putamen, insula, and cerebellum areas that also are found in task-related sensorimotor SM reference, see the red color areas in Fig. 8(4).

**Fig. 9.**
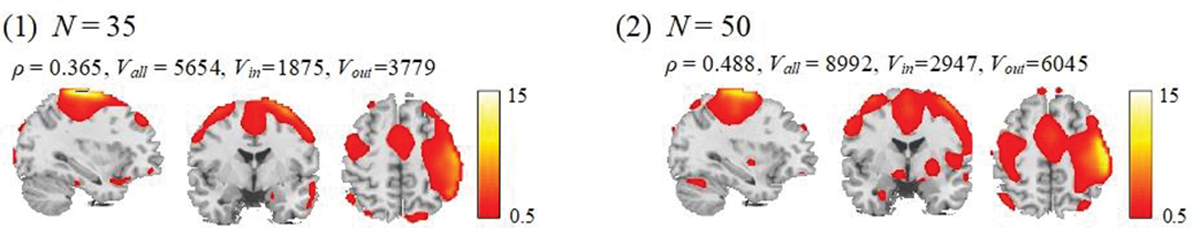
The shared sensorimotor SM estimates by real-valued T-sICA for the magnitude-only residual fMRI data after filtering under two differences number of components *N* : (1) *N* = 35; (2) *N* = 50. The *ρ*, *V*_*all*_, *V*_*in*_, and *V*_*out*_ values are calculated.

In addition, since residual fMRI data after filtering are very noisy, we conduct real-valued T-sICA on the magnitude of residual fMRI data after filtering as shown in Fig. 9 to inspect the missing information for filtered fMRI data. Though the shared SMs extracted by T-sICA at *N* = 35 and *N* = 50 are nosier and have lower *ρ* and *V*_*in*_ values than ICA-sCPD in magnitude-only raw fMRI data analysis (see Fig. 9), the shared SMs still show obvious activations in RPMA, SMA, and LPMA areas. Moreover, the shared SM at *N* = 50 has higher *ρ* and *V*_*in*_ values as well as extracts more meaningful activations in RPMA, SMA, and LPMA areas than at *N* = 35. This indicates the residual fMRI data after filtering require higher number of components to extract interested components.

## V. Conclusion and Discussion

In the framework of AR_R_R_1_LS, the proposed constrained sAR_R_R_1_LS-PO method takes advantage of temporal shift-invariant multiway structure, the spatial orthonormality, and the small spatial source phase character of BOLD-related voxels to simultaneously cope with high-noisy nature and inter-subject spatiotemporal variability of complex-valued multi-subject fMRI data. We perform both simulated and experimental fMRI data experiments, and conclude that AR_R_R_1_LS shows better separation performance than several state-of-the-art CPD methods, and the proposed sAR_R_R_1_LS-PO shows improved estimates of task-related sensorimotor and auditory components than pcsCPD and T-sICA, and extracts better sensorimotor components for noisier but intact raw fMRI data than for filtered fMRI data. Furthermore, the complex-valued sAR_R_R_1_LS-PO extracts 30.7% more interesting activated voxels than real-valued ICA-sCPD in the magnitude-only fMRI data analysis.

Compare with sAR_*R*_R_1_LS-PO and pcsCPD, T-sICA shows the worst sensorimotor shared SM and TC estimates for both simulated and experimental fMRI data analyses. The reason is that T-sICA combines two different cost functions (i.e., ICA and rank-1 ALS) which may lead to divergence. Moreover, the separation performance of T-sICA relies on ICA. However, the AR_*R*_R_1_LS used in sAR_*R*_R_1_LS-PO which minimizes the squares of errors for each iteration to avoid algorithm divergence. On the other side, the pcsCPD utilizes rank-*R* ALS to update all loading matrices, i.e., shared SMs, shared TCs, subject-specific time delays and intensities. Due to incorporating the shift-invariance and spatial phase sparsity constraint, pcsCPD shows better separation performance than T-sICA in terms of sensorimotor shared SMs and TCs as shown in Figs. 5~7 and auditory shared SMs and TCs in filtered fMRI data analysis as shown in Figs. S3~S5 (see supplementary materials for details). However, pcsCPD gets obviously declined separation performance of auditory component in nosier raw fMRI data analysis than in filtered fMRI data analysis. Moreover, the lower computation complexity of the proposed method than pcsCPD is discussed in supplementary materials. Meanwhile, the accALS and enALS based on rank-*R* ALS exhibit worse separation performance than AR_*R*_R_1_LS as shown in Fig. S1 and Table SI. This implies that the AR_*R*_R_1_LS can relax the strict CPD model and is more appropriate for tensors that do not well conform CPD model.

The excellent efficacy of the small phase property of BOLD- related voxels and spatial orthogonality for complex-valued fMRI data are further demonstrated in this paper. In fact, we conducted ablation experiments on sAR_*R*_R_1_LS-PO, and concluded that the spatial phase sparsity constraint and spatial orthonormality constraint both obviously improve the separation performance and the combination of these two constraints can further promote the separation performance. Since the complex-valued shared SMs contain huge number of unwanted high-magnitude noise voxels. Therefore, directly incorporating sparsity on magnitude part of shared SMs is not suggested. Thanks to the small phase property of BOLD-related voxels, we can gradually add sparsity on the voxels that have large phase values after phase de-ambiguity for each iteration. The separation improvement of the proposed sAR_*R*_R_1_LS-PO and pcsCPD attributes to the phase sparsity constraint, which facilitates sparse representation of complex-valued fMRI data. Besides, we further verify improved performance of proposed method with orthonormality than without orthonormality especially for raw fMRI data (see supplementary materials). Due to allowing subject-specific time delays, the proposed sAR_*R*_R_1_LS-PO and pcsCPD all present higher average *ρ* values of sensorimotor and auditory shared TCs than T-sICA in both raw and filtered fMRI data analyses (see Figs. 5 and S3). Besides, sAR_*R*_R_1_LS-PO can more accurately estimate time delays than pcsCPD (see Figs. 7(3) and S5(3)), especially for time delay equaling to 0 and large values for both sensorimotor and auditory components.

The pcsCPD utilizes the phase de-ambiguity method based on the maximization of the real-part power of each column of aggregating mixing matrix. However, the length of aggregating mixing matrix is generally longer than the number of time points (*JK* vs. *J*). This may lead to obtain a wrong angle for phase de-ambiguity. Therefore, we here propose an accurate phase de-ambiguity method based on the maximal correlation coefficient between the phase-rotated shared SM component and its magnitude. We also compared these two phase de-ambiguity methods on sAR_*R*_R_1_LS-PO in raw and filtered fMRI data analyses, and summarized better performance of the proposed accurate phase de-ambiguity method.

Due to the lower noise level of filtered fMRI data than raw fMRI data, ICA-sCPD and pcsCPD all show better sensorimotor and auditory shared SM and TC estimates in filtered fMRI data analysis than in raw fMRI data analysis. However, some meaningful information is also removed for the filtered fMRI data. We can see from Fig. 9 that the interesting LPMA, RPMA, and SMA of sensorimotor shared SM estimates can be extracted when conducting real-valued T-sICA on residual fMRI data after filtering. Fortunately, on the premise of the best separation performance, the proposed sAR_*R*_R_1_LS-PO in raw fMRI data analysis acquires not only higher *ρ* values of task-related sensorimotor shared SMs and TCs but also more meaningful task-related sensorimotor contiguous activations than those in filtered fMRI data analysis (see Figs. 5~7 and Table I). Therefore, the proposed sAR_*R*_R_1_LS-PO is robust to noise and can well extract the task-related sensorimotor contiguous activations even for noisy complex-valued fMRI data. In addition, compared with ICA-sCPD in magnitude-only analysis, sAR_*R*_R_1_LS-PO can additionally extract 30.7% more useful interesting activations in complex-valued analysis. In practice, subject-specific components are often needed in addition to the shared components in the analysis of multi-subject fMRI data. For example, a shared and subject-specific dictionary learning method simultaneously extracts shared and subject-specific components [12] by using intrinsic spatial sparsity of fMRI data [11]. In the future, we will apply back-projection [34] to the proposed method to also extract the subject-specific information, taking advantages of efficient shift-invariance, spatial orthonormality and phase sparsity constraints. In consider of satisfying separation performance of task-related fMRI data, we will apply the proposed method to resting-state fMRI data in the future work and try to classify healthy controls and patients of brain disease by exploiting complex-valued shared spatial and temporal features [35], [36] extracted by the proposed method.

## Supplementary Material

supp1-3198679

## Figures and Tables

**TABLE I:** Comparison of T-sICA, pcsCPD, and sAR_*R*_R_1_ LS-PO for Analyzing the Experimental Raw and Filtered Complex-Valued Multi-Subject fMRI Data in Terms of *V_all_*, *V_in_*, *V_out_* and *V_in_*/*V_all_* of Shared SMs Estimates in Fig. 6. The Bold Values Are the Largest Values OF *V_all_*, *V_in_*, AND *V_in_*/*V_all_* AND THE LOWEST *V_out_* for Each Case

	Raw fMRI data analysis				Filtered fMRI data analysis			
	*V_all_*	*V_in_*	*V_out_*	*V_in_*/*V_all_*	*V_all_*	*V_in_*	*V_out_*	*V_in_*/*V_all_*
		
T-sICA	3631	1254	**2377**	0.345	5277	2319	**2958**	0.439
pcsCPD	6010	1805	4205	0.300	**8910**	3437	5473	0.386
sARbRiLS-PO	**9335**	**4136**	5199	**0.443**	8559	**3788**	4771	**0.443**
