# Supplementary material for: Constrained CPD of Complex-Valued Multi-Subject fMRI Data via Alternating Rank-R and Rank-1 Least Squares
Source: IEEE Trans Neural Syst Rehabil Eng. Author manuscript; Available in PMC 2022 Oct 28. (PMC9613874; doi:10.1109/TNSRE.2022.3198679)
Supplement: supp1-3198679 [file NIHMS1837383-supplement-supp1-3198679.pdf]

Constrained CPD of Complex-Valued Multi-Subject fMRI Data via Alternating Rank-*R* and Rank-1 Least Squares

Li-Dan Kuang, *Member, IEEE,* Qiu-Hua Lin, *Member, IEEE,* Xiao-Feng Gong, *Member, IEEE,* Jianming Zhang, *Member, IEEE,* Wenjun Li, Feng Li, and Vince D. Calhoun, *IEEE Fellow*

Supplementary materials

A. Derivation of (24) from (23)

We respectively expand two terms in (23) as follows:

$$\begin{aligned}&\mathrm{Re}\{\mathbf{m}_{r,k}^T\}\,\mathrm{Re}\{\mathbf{b}_r c_{k,r}\}\\&=\mathrm{Re}\{c_{k,r}\}\sum_{j=1}^{J}\mathrm{Re}\{b_r(j-\tau_{k,r})\}\,\mathrm{Re}\{m_{r,k}(j)\},\end{aligned}\quad (\text{S.1})$$

$$\begin{aligned}&\mathrm{Im}\{\mathbf{m}_{r,k}^T\}\mathrm{Im}\{\mathbf{b}_r c_{k,r}\}\\&=\mathrm{Im}\{c_{k,r}\}\sum_{j=1}^{J}\mathrm{Im}\{b_r(j-\tau_{k,r})\}\mathrm{Im}\{m_{r,k}(j)\}\end{aligned}.\quad (\text{S.2})$$

We can calculate the above two terms in the frequency domain, that is,

$$\begin{aligned}&\sum_{j=1}^{J}\mathrm{Re}\{b_r(j-\tau_{k,r})\}\,\mathrm{Re}\{m_{r,k}(j)\}\\&\rightarrow\tilde{\phi}_1(f)=(\tilde{\mathrm{R}}\mathrm{e}\{m_{r,k}\}(f))^*\,\tilde{\mathrm{R}}\mathrm{e}\{b_r\}(f)\end{aligned},\quad (\text{S.3})$$

$$\begin{aligned}&\sum_{j=1}^{J}\mathrm{Im}\{b_r(j-\tau_{k,r})\}\,\mathrm{Im}\{m_{r,k}(j)\}\\&\rightarrow\tilde{\phi}_2(f)=(\tilde{\mathrm{I}}\mathrm{m}\{m_{r,k}\}(f))^*\,\tilde{\mathrm{I}}\mathrm{m}\{b_r\}(f)\end{aligned},\quad (\text{S.4})$$

where $\tilde{\phi}_1(f)$, $\tilde{\phi}_2(f)$, $\tilde{\mathrm{R}}\mathrm{e}\{m_{r,k}\}(f)$, $\tilde{\mathrm{I}}\mathrm{m}\{m_{r,k}\}(f)$, $\tilde{\mathrm{R}}\mathrm{e}\{b_r\}(f)$, and $\tilde{\mathrm{I}}\mathrm{m}\{b_r\}(f)$ are the elements of $\tilde{\boldsymbol{\phi}}_1$, $\tilde{\boldsymbol{\phi}}_2$, $\tilde{\mathrm{R}}\mathrm{e}\{\mathbf{m}_{r,k}\}$, $\tilde{\mathrm{I}}\mathrm{m}\{\mathbf{m}_{r,k}\}$, $\tilde{\mathrm{R}}\mathrm{e}\{\mathbf{b}_r\}$, and $\tilde{\mathrm{I}}\mathrm{m}\{\mathbf{b}_r\}$, respectively. $f=1,\cdots,F$. We then transform $\tilde{\boldsymbol{\phi}}_1$ and $\tilde{\boldsymbol{\phi}}_2$ into the time domain as $\boldsymbol{\phi}_1=\{\phi_1(j)\}\in\mathbb{C}^J$ and $\boldsymbol{\phi}_2=\{\phi_2(j)\}\in\mathbb{C}^J$. The sum of two terms in (21) for time point *j* becomes

$$\varphi_{k,r}(j)=|\,\mathrm{Re}\{c_{k,r}\}\phi_1(j)\,|+|\,\mathrm{Im}\{c_{k,r}\}\phi_2(j)\,|.\quad (\text{S.5})$$

Thus, the time delay $\tau_{k,r}$ can be obtained by maximizing $\varphi_{k,r}(j)$, $j=1,\ldots,J$:

$$\hat{\tau}_{k,r}=\arg\max_{1\le j\le J}\varphi_{k,r}(j),\ \ \tau_{k,r}=\hat{\tau}_{k,r}-J+1.\quad (\text{S.6})$$

B. Comparison Results of AR$_R$R$_1$LS and Several CPD Methods

In order to inspect the advantage of the proposed AR$_R$R$_1$LS for decomposing multi-subject fMRI data, we compare AR$_R$R$_1$LS with several popular CPD methods: (1) accALS which shows similar separation performance with ALS but is more faster than ALS [S1]; (2) ALS with enhanced linear search (enALS) [S2], a hybrid between alternating optimization and ADMM (AO-ADMM) [S3]; (3) nonlinear least squares (NLS) [S4, S5]; (4) minimizing a function by L-BFGS with dogleg trust region (MINF) [S4, S5]; (5) COMFAC [S6]. Other CPD methods such as flexible and fast algorithm for the CP decomposition (FFCP) [S9], CPD via proximal alternating linearized minimization (PALS) [S10], Krylov-Levenberg-Marquardt (KLM) [S11], Nesterov-based alternating optimization [S12], and CPD based on stochastic proximal gradient optimization including block-randomized stochastic gradient descent (SGD) for CPD (BrasCPD) [S7], Adagrad version of block-randomized CPD (AdaCPD) [SS7], and accelerated stochastic CPD (ASCPD) [S8] all show unsatisfying separation performance for simulated and experimental multi-subject fMRI data, thus we do not show the detailed comparison results. We set $\varepsilon_{\text{iter\_min}}=10^{-6}$ and iter$_{\max}$ = 200 for each method. We repeat each algorithm 20 times for each case. We next examine the separation performance of AR$_R$R$_1$LS, accALS, enALS, AO-ADMM, NLS, MINF, and COMFAC in both simulated and actual fMRI data analyses.

1) Simulated fMRI data analysis

We examine noise effects of different SNR levels on AR$_R$R$_1$LS with several CPD algorithms (i.e., accALS, enALS, AO-ADMM, NLS, MINF, and COMFAC) under simulated fMRI data with spatial change, as shown in Fig. S1. Note, since these CPD methods do not incorporate the shift-invariance property, we here do not consider the temporal time delay differences. We can conclude from Fig. S1 that the proposed AR$_R$R$_1$LS has the highest average ρ values of task-related shared SMs and TCs for all SNR levels. Moreover, AR$_R$R$_1$LS gets obviously higher average ρ values of shared TCs when lower SNR levels ≤ –18 dB. As a whole, MINF ranks the last. COMFAC shows the lowest average ρ values of shared SMs when SNR ≤ –18 dB and the second lowest average ρ values of shared TCs when –16 dB ≤ SNR ≤ –10 dB. Due to using the enhanced linear search, enALS generally gets better average ρ values than accALS. Compared with enALS and accALS, NLS and AO-ADMM display higher average ρ values of shared SMs when larger SNR levels ≥ –6 dB, but obtains lower average ρ values of shared TCs when smaller SNR levels (i.e., –16 dB ≤ SNR ≤ –12 dB).

2) Actual fMRI data analysis

In order to further show the outperformed separation performance of AR$_R$R$_1$LS, we present Table SI to show

average ρ values of task-related sensorimotor shared SMs and TCs extracted by accALS, COMFAC, AO-ADMM, NLS, MINF, enhanced ALS, and AR$_R$R$_1$LS in both experimental raw and filtered fMRI data analyses. The AR$_R$R$_1$LS obviously shows the highest average ρ values of shared SMs and TCs in both raw and filtered fMRI data analyses. The accALS and enhanced ALS exhibit similar average ρ values of shared SMs and TCs in raw and filtered fMRI data analyses. This indicates the enhanced line search may do not well work for high-noisy complex-valued fMRI data with high inter-subject variability. COMFAC gets close average ρ values of shared SMs to accALS, while obtains slightly lower average ρ values of shared TCs than accALS. In terms of average ρ values of shared SMs and TCs, NLS, AO-ADMM, and MINF are slightly higher than accALS and enhanced ALS in raw fMRI data analysis, but are lower than accALS and enhanced ALS in filtered fMRI data analysis. These seven methods all show obviously higher average ρ values of shared SM and TC estimates in filtered fMRI data analysis than nosier raw fMRI data analysis.

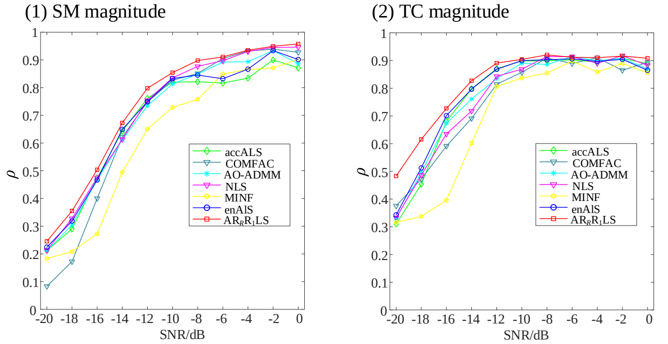

Fig. S1. Comparison of the noise effects (from SNR = –20 dB to SNR = 0 dB with interval 2 dB) on the accALS, COMFAC, AO-ADMM, NLS, MINF, enALS, and AR$_R$R$_1$LS in terms of the average ρ values between the task-related shared SM magnitude (1) and TC magnitude (2) estimates and their ground truths. The true component number N = 30 is used.

TABLE SI
COMPARISON OF ACCALS, COMFAC, AO-ADMM, NLS, MINF, ENALS, AND AR$_R$R$_1$LS FOR ANALYZING THE EXPERIMENTAL RAW AND FILTERED COMPLEX-VALUED MULTI-SUBJECT FMRI DATA OVER 20 RUNS IN TERMS OF AVERAGE ρ VALUES OF TASK-RELATED SENSORIMOTOR SM AND TC MAGNITUDE ESTIMATES. THE LARGEST ρ VALUES ARE BOLD FOR EACH CASE.

	Raw fMRI data		Filtered fMRI data	
	SM	TC	SM	TC
accALS	0.082	0.258	0.160	0.439
COMFAC	0.082	0.246	0.162	0.390
AO-ADMM	0.135	0.316	0.152	0.289
NLS	0.083	0.280	0.111	0.380
MINF	0.130	0.279	0.121	0.312
enALS	0.080	0.258	0.163	0.428
AR$_R$R$_1$LS	**0.243**	**0.463**	**0.286**	**0.668**

C. *Results of Task-related Auditory Component*

For the experimental fMRI data, another task-related component is auditory component. Thus we further present the comparison results of sAR$_R$R$_1$LS-PO, T-sICA, and pcsCPD in this subsection. Since group ICA is widely-acceptable for multi-subject fMRI data analysis and magnitude-only fMRI data persist smaller noise than complex-valued fMRI data, we use auditory SM extracted by group ICA [S13] of magnitude-only multi-subject fMRI data as the auditory SM reference in Fig. S2(a). Besides, we generate the task-related auditory TC references by convolving the stimuli with the canonical SPM hemodynamic response functions in Fig. S2(b).

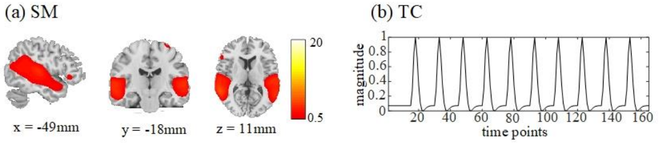

Fig. S2. The SM and TC references of task-related auditory component for experimental fMRI data. The auditory SM reference (a) is the SM estimated by widely-used group ICA of magnitude-only fMRI data. The TC references (b) are obtained by convolving the stimuli with the canonical SPM hemodynamic response functions.

We further exhibit Fig. S3 to show means and standard deviations of ρ values of typical auditory component of T-sICA, pcsCPD, and sAR$_R$R$_1$LS-PO. The magnitude and phase parts of auditory shared SMs and TCs are showed. As expected, sAR$_R$R$_1$LS-PO still acquires the highest average ρ values in all cases in Fig. S3. For the average ρ values of auditory shared SMs and TCs in Fig. S3, pcsCPD is higher than T-sICA in filtered fMRI data analysis but is lower than T-sICA in raw fMRI data analysis. This indicates that pcsCPD is sensitive to noise when extracting auditory component. Moreover, as the auditory component is more difficult to decompose than sensorimotor component, these three methods all show lower average ρ values of auditory shared SM and TC estimates in noisier raw fMRI data analysis than in filtered fMRI data analysis. In addition, these three methods comprehensively obtain the larger standard deviations of ρ values in raw fMRI data analysis than in filtered fMRI data analysis as shown in Fig. S3.

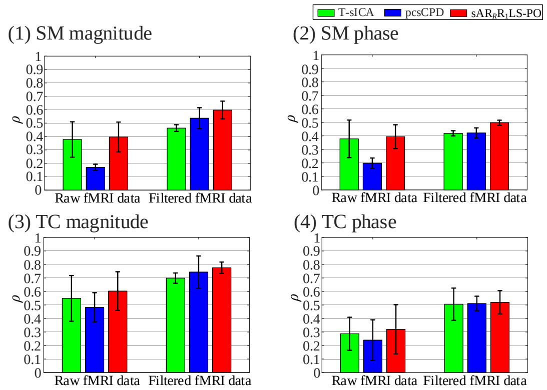

Fig. S3. Comparison of T-sICA, pcsCPD, and sAR$_R$R$_1$LS-PO for analyzing the experimental raw and filtered complex-valued multi-subject fMRI data over 20 runs in terms of the means and standard deviations of ρ values for the auditory shared SM magnitude (1), SM phase (2), TC magnitude (3), and TC phase (4) estimates.

We also present Fig. S4 to show magnitude and phase parts of the typical auditory shared SMs estimated by T-sICA, ICA-sCPD, pcsCPD, and sAR$_R$R$_1$LS-PO in raw and filtered fMRI data analyses. The V_{all}, V_{in}, V_{out}, and V_{in}/V_{all} values of typical sAR$_R$R$_1$LS-PO has the smallest noise voxels (i.e., smallest V_{out}

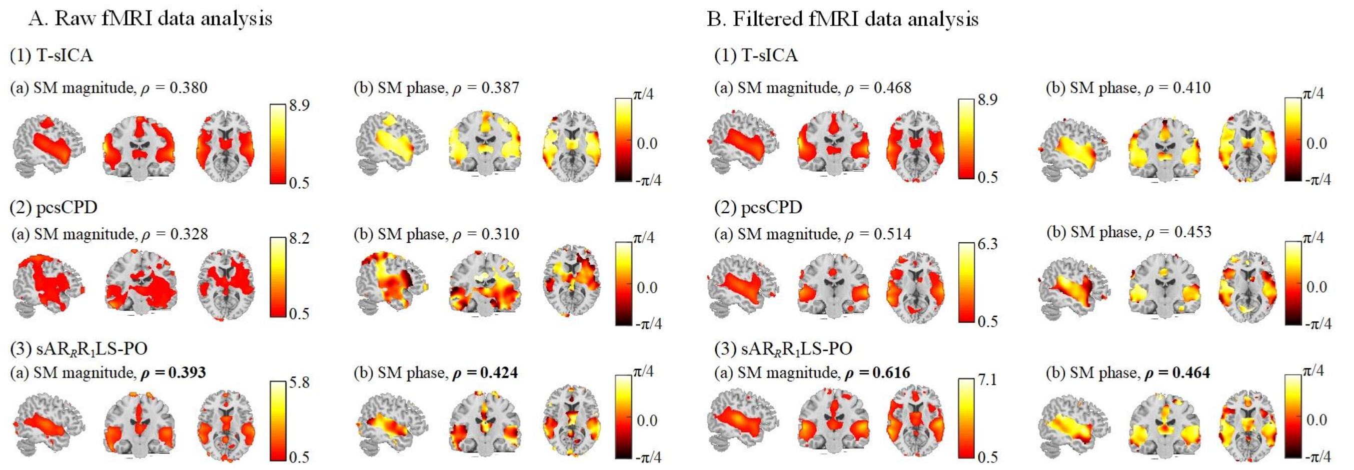

Fig. S4. Typical auditory shared SMs estimated by T-sICA, pcsCPD, and sAR$_R$R$_1$LS-PO for analyzing the experimental raw (A) and filtered (B) complex-valued multi-subject fMRI data. The magnitude (a) and phase images (b) of shared SMs and corresponding ρ values are showed. The largest ρ values are bold. The phase correction and de-ambiguity are performed on shared SMs, and thus the phase values of phase maps range from –π/4 to π/4.

values) and the highest V_{in} and V_{in}/V_{all} values reflecting in left shared SMs in these two cases are displayed in Table SII. From Fig. S4 and Table SII, the shared SMs estimated by proposed and right middle, superior and inferior temporal areas middle, superior and inferior temporal areas in both two analyses. We can conclude that these three methods relatively obtain better shared SMs (i.e., higher ρ values and V_{in} values) in filtered fMRI data analysis than in raw fMRI data analysis. The pcsCPD extracts better shared SMs (higher ρ, V_{in}, and V_{in}/V_{all} values and lower V_{out} value) than T-sICA in filtered fMRI data analysis, while obtains the worst shared SMs in nosier raw fMRI data analysis (see Fig. S4 and Table SII).

TABLE SII
COMPARISON OF T-SICA, PCSCPD, AND SAR$_R$R$_1$LS-PO FOR ANALYZING EXPERIMENTAL RAW AND FILTERED COMPLEX-VALUED MULTI-SUBJECT FMRI DATA IN TERMS OF V_{all}, V_{in}, V_{out} AND V_{in}/V_{all} OF SHARED SMS ESTIMATES IN FIG. S4. THE BOLD VALUES ARE THE LARGEST VALUES OF V_{all}, V_{in}, AND V_{in}/V_{all} AND THE LOWEST V_{out} FOR EACH CASE.

	Raw fMRI data analysis				Filtered fMRI data analysis			
	V_{all}	V_{in}	V_{out}	V_{in}/V_{all}	V_{all}	V_{in}	V_{out}	V_{in}/V_{all}
T-sICA	10541	2649	7892	0.251	**11815**	2924	8891	0.248
pcsCPD	**11528**	1797	9731	0.156	10855	2990	7865	0.275
sAR$_R$R$_1$LS-PO	9867	**2701**	**7166**	**0.274**	10969	**3228**	**7741**	**0.294**

For auditory component, we give Fig. S5 to present the results of shared TC magnitude and phase parts, subject-specific time delays and intensities in raw and filtered fMRI data analyses. The magnitude and phase parts of shared TCs estimated by the proposed sAR$_R$R$_1$LS-PO can better follow fluctuations of auditory TC reference than other two methods in both two cases in Figs. S5(1)~(2). Moreover, the number of correctly estimated time delays of the proposed sAR$_R$R$_1$LS-PO is larger than pcsCPD as shown in Fig. S5(3). More specifically, the sAR$_R$R$_1$LS-PO not only can accurately estimate the larger time delay (e.g., subject 1), but also can accurately estimate the zero time delays. Furthermore, subject-specific intensities curves of pcsCPD are closer to those of sAR$_R$R$_1$LS-PO than those of T-sICA in both two analyses (see Fig. S5(4)). More specifically, the ρ values are 0.824 vs. 0.718 in raw fMRI data analysis and 0.924 vs. 0.853 in filtered fMRI data analysis.

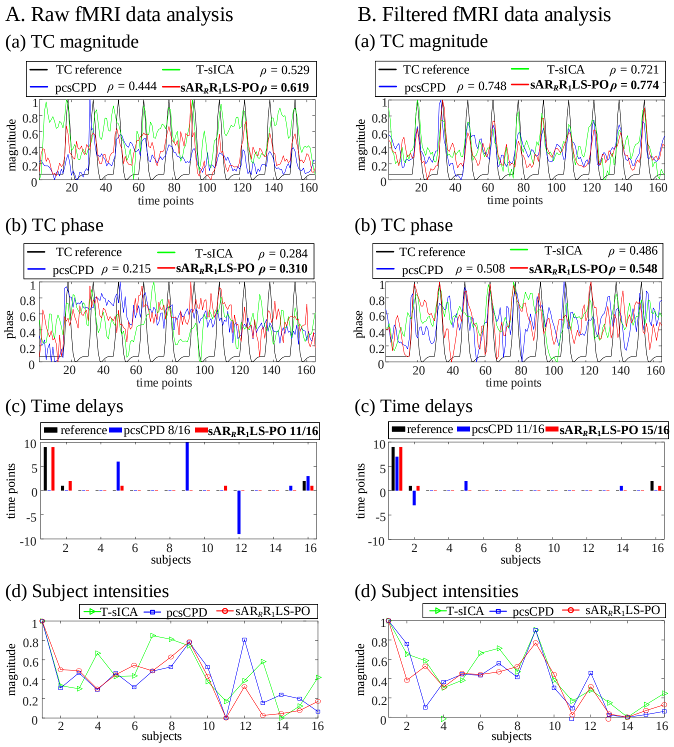

Fig. S5. Comparison of T-sICA, pcsCPD, and sAR$_R$R$_1$LS-PO for analyzing the experimental raw (A) and filtered (B) complex-valued multi-subject fMRI data in terms of typical auditory shared TC magnitude parts (1), shared TC phase parts (2), time delays (3) and subject intensities (4). The ρ values of shared TC magnitude and phase parts and the number of correct time delays are calculated. The largest ρ value and the highest number of correct time delays for each case are bold.

D. *Comparison Results of Orthonormality Constraint*

In this subsection, in order to evaluate the importance of orthonormality of the proposed method, we further evaluate the separation performance of the proposed method without orthonormality constraint (shorted as sAR$_R$R$_1$LS-P) and with orthonormality constraint (i.e., sAR$_R$R$_1$LS-PO) as show in Table SIII. As for the raw fMRI data analysis, sAR$_R$R$_1$LS-PO shows obviously higher means and lower standard deviations

of ρ values of sensorimotor shared SM magnitude, SM phase, TC magnitude, and TC phase estimates than sAR$_R$R$_1$LS-P. In the filtered fMRI data analysis, sAR$_R$R$_1$LS-PO also shows higher average ρ values than sAR$_R$R$_1$LS-P. However, the ρ value differences between sAR$_R$R$_1$LS-PO and sAR$_R$R$_1$LS-P in filtered fMRI data analysis are smaller than in raw fMRI data analysis. This indicates that the orthonormality constraint can effectively improve the performance of the proposed method for noisy raw complex-valued fMRI data analysis.

TABLE SIII
COMPARISON OF sAR$_R$R$_1$LS-P AND sAR$_R$R$_1$LS-PO FOR ACTUAL RAW AND FILTERED COMPLEX-VALUED fMRI DATA IN TERMS OF THE MEANS AND STANDARD DEVIATIONS OF ρ VALUES FOR THE TASK-RELATED SENSORIMOTOR SHARED SM MAGNITUDE, SM PHASE, TC MAGNITUDE, AND TC PHASE ESTIMATES. THE MAXIMUM MEANS AND MINIMUM STANDARD DEVIATIONS OF ρ VALUES ARE BOLD.

	Raw fMRI data		Filtered fMRI data	
	sAR$_R$R$_1$LS-P	sAR$_R$R$_1$LS-PO	sAR$_R$R$_1$LS-P	sAR$_R$R$_1$LS-PO
SM magnitude	0.557±0.182	**0.692±0.013**	0.586±0.044	**0.631±0.036**
SM phase	0.458±0.096	**0.543±0.013**	0.462±**0.027**	**0.511**±0.040
TC magnitude	0.897±0.135	**0.913±0.019**	0.880±**0.028**	**0.881**±0.042
TC phase	0.527±0.281	**0.642±0.178**	0.607±0.255	**0.662±0.172**

E. Comparison Results of Computation Cost

We finally analyze the computation cost of the proposed sAR$_R$R$_1$LS-PO method with other methods from the following aspects: computation complexity, computation time and maximum computation memory for actual fMRI data experiment. First, we compare the computation complexities of updating shared SMs **S**, shared TCs **B** and subject-specific intensities **C** for the proposed method and pcsCPD, as shown in Table SIV. The complexity of the proposed method is approximately R/V^3 of that of pcsCPD. Since $R<<V$ (i.e., R = 50 vs. V = 59610 in actual fMRI experiment), the proposed method has much lower computation complexity than pcsCPD.

TABLE SIV
COMPUTATION COMPLEXITY COMPARISON OF sAR$_R$R$_1$LS-PO AND pcsCPD IN TERMS OF UPDATING SHARED SMs **S**, SHARED TCs **B** AND SUBJECT-SPECIFIC INTENSITIES **C** FOR EACH ITERATION.

	pcsCPD	sAR$_R$R$_1$LS-PO
updating **S**	$\mathcal{O}\left(VJKR+JKR+J^3K^3\right)$	$\mathcal{O}\left(VJKR+JKR+J^3K^3\right)$
updating **B**	$\mathcal{O}\left(VJKR+VKR+V^3K^3\right)$	$\mathcal{O}\left(JKR+K^3R\right)$
updating **C**	$\mathcal{O}\left(VJKR+VJR+V^3J^3\right)$	$\mathcal{O}\left(JKR+J^3R\right)$

TABLE SV
COMPUTATION TIME AND MAXIMUM MEMORY COMPARISONS OF T-sICA, pcsCPD AND sAR$_R$R$_1$LS-PO FOR ACTUAL fMRI ANALYSIS. WE SET THE NUMBER OF MAXIMUM ITERATIONS OF pcsCPD AND sAR$_R$R$_1$LS-PO AS 200. "MB" AND "s" RESPECTIVELY DENOTE MEGABYTE AND SECOND.

	T-sICA	pcsCPD	sAR$_R$R$_1$LS-PO
Computation time	5,500.0MB	18,178.5MB	6531.4MB
Computation memory	1,492.8s	41,235.1s	5,140.1s

Second, we illustrate the computation time and maximum computation memory for T-sICA, pcsCPD, and sAR$_R$R$_1$LS-PO in Table SV. Notice that we run the methods on MATLAB2020a under Win10 operating system with Intel(R) Core(TM) i7-8700 CPU @ 3.20GHz and 32G memory. Although T-sICA costs the minimum computation time and memory, it shows the worst separation performance for both simulated and experimental fMRI data analyses (see Figs. 3 and 5), compared with pcsCPD and sAR$_R$R$_1$LS-PO. The proposed sAR$_R$R$_1$LS-PO requires 702.2% lower computation time and 178.3% lower computation memory than pcsCPD. Above all, the proposed method not only shows better separation performance but also owns lower computation complexity and higher speed than pcsCPD.

REFERENCES

[S1] G. Tomasi. Practical and computational aspects in chemometric data analysis. Ph.D. thesis, The Royal Veterinary and Agricultural University, Frederiksberg, Denmark, 2006.

[S2] L. Sorber, I. Domanov, M. Van Barel, and L. De Lathauwer, "Exact line and plane search for tensor optimization," *Computational Optimization and Applications*, vol. 63, no. 1, pp. 121–142, Jan. 2016.

[S3] K. Huang, N.D. Sidiropoulos, and A. P. Liavas, "A flexible and efficient algorithmic framework for constrained matrix and tensor factorization," *IEEE Transactions on Signal Processing*, vol. 64, no. 19, pp. 5052–5065, Oct. 2016.

[S4] L. Sorber, M.V. Barel, and L.D. Lathauwer, "Unconstrained optimization of real functions in complex variables," *SIAM Journal on Optimization*, vol. 22, no. 3, pp. 879–898, Jan. 2012.

[S5] L. Sorber, M. Van Barel, and L. De Lathauwer, "Optimization-based algorithms for tensor decompositions: Canonical polyadic decomposition, decomposition in rank-(Lr,Lr,1) terms, and a new generalization," *SIAM Journal on Optimization*, vol. 23, no. 2, pp. 695–720, Jan. 2013.

[S6] N.D. Sidiropoulos, G.B. Giannakis, and R. Bro, "Blind PARAFAC receivers for DS-CDMA systems," *IEEE Transactions on Signal Processing*, vol. 48, no. 3, pp. 810–823, Mar. 2000.

[S7] X. Fu, S. Ibrahim, H.-T. Wai, C. Gao, and K. Huang, "Block-randomized stochastic proximal gradient for low-rank tensor factorization," *IEEE Transactions on Signal Processing*, vol. 68, pp. 2170–2185, 2020.

[S8] I. Siaminou and A.P. Liavas, "An accelerated stochastic gradient for canonical polyadic decomposition," *arXiv:2109.13964*, Sep. 2021, [Online]. Available: http://arxiv.org/abs/2109.13964.

[S9] Y. Qiu, G. Zhou, Y. Zhang, and A. Cichocki, "Canonical polyadic decomposition (CPD) of big tensors with low multilinear rank," *Multimedia Tools and Applications*, vol. 80, no. 15, pp. 22987–23007, Jun. 2021.

[S10] J. Bolte, S. Sabach, and M. Teboulle, "Proximal alternating linearized minimization for nonconvex and nonsmooth problems," *Mathematical Programming*, vol. 146, no. 1–2, pp. 459–494, Aug. 2014.

[S11] P. Tichavský, A.H. Phan, and A. Cichocki, "Krylov-levenberg-marquardt algorithm for structured tucker tensor decompositions," *IEEE Journal of Selected Topics in Signal Processing*, vol. 15, no. 3, pp. 550–559, Apr. 2021.

[S12] A.P. Liavas, G. Kostoulas, G. Lourakis, K. Huang, and N.D. Sidiropoulos, "Nesterov-based alternating optimization for nonnegative tensor factorization: Algorithm and parallel implementation," *IEEE Transactions on Signal Processing*, vol. 66, no. 4, pp. 944–953, Feb. 2018.

[S13] V.D. Calhoun, T. Adali, G.D. Pearlson, and J.J. Pekar, "A method for making group inferences from functional MRI data using independent component analysis," *Human Brain Mapping*, vol. 14, no. 3, pp. 140–151, Nov. 2001.
